# Recovery of hydrothermal wustite-magnetite spherules from the Central Indian Ridge, Indian Ocean

**DOI:** 10.1038/s41598-022-10756-1

**Published:** 2022-04-26

**Authors:** Deepak K. Agarwal, John Kurian Palayil

**Affiliations:** National Centre for Polar and Ocean Research, Ministry of Earth Sciences, Headland Sada, Vasco-da-Gama, Goa 403804 India

**Keywords:** Ocean sciences, Geochemistry

## Abstract

A sediment sample with high abundance (19 spherules in ~ 85 g) of spherules was recovered from Central Indian Ridge (CIR) segment S2 (70° 54′ E, 25° 14′ S to 70° 50′ E, 24° 41′ S), ~ 85 km north of Rodrigues triple junction (RTJ). On the external surface of the spherules, magnetite appears as crystals, whereas wustite mostly appears as a homogenous glass phase. These spherules are composed of wustite and magnetite hosting Mn, unlike micrometeorites which essentially host Ni. Mn is more heterogeneously distributed with a relatively higher concentration in the wustite phase than the magnetite, suggesting hydrothermal origin. Furthermore, the presence of sulfide nano-particles in the wustite phase and a minor quantity of Pb and S in the ferrihydrite matrix points to the fact that CIR spherules are of hydrothermal origin. The CIR spherules could have formed either by the interaction of the reduced hydrothermal fluids with the ultramafic/basaltic rocks or silica-undersaturated magmatic melts, or by alteration of original particles (such as cosmic spherules, volcanic spherules, or even foraminifera) via Mn-bearing hydrothermal fluids, such as released during the serpentinization of ultramafic rocks. The finding of Mn hosting wustite-magnetite assemblage suggests an hydrothermal system in the near vicinity and can be considered as an additional proxy for indication of hydrothermal activity.

## Introduction

Spherules of different elemental compositions such as silicate spherule^1^, aluminum spherule^2^, metallic Fe-rich (magnetite/ wustite) spherules^3^ have been reported from cosmic/ extraterrestrial materials as well as from a variety of different geological environments like deep-ocean sediments, polar ice, Paleozoic salt deposits, and sedimentary rocks as old as Archean (> 2.5 Ga)^4–10^. Among these, Fe-rich spherules of extraterrestrial origin are widely investigated to establish the flux of extraterrestrial dust to Earth (Prasad et al.^8^ and references therein). Earlier studies on cosmic spherules have suggested three types: the chondritic stony spherules (type-S), the glass spherules with dendritic magnetite (type-G), and the non-chondritic iron spherules (type-I)^7,11^. Several criteria have been proposed for the identification of the type-I cosmic spherules: (1) morphological features like spherical shape, dendritic phases, and void spaces; (2) chemical signatures like Ni-bearing wustite/magnetite, FeNi metal, and particles of MET (metal bead-bearing spherules), OXMET (these particles contain metal within an oxide mantle containing wustite and magnetite), and OX (metal bead-free spherules) type^3^. The type-I or Fe-rich non-cosmic spherules formed in situ on Earth may also possess similar morphological features like spherical shape, dendritic phases, and void spaces^6^. On Earth, spherules can form by biological activity, from molten droplets in a magmatic/hydrothermal environment, and diagenetic activity forming magnetite framboids by replacing pyrite^12,13^.

Iyer et al.^14^ suggested that Fe-rich lavas or hydrothermal emanations reacting with siliceous ooze can also form spherules and compared them to experimentally produced spherules. Under hydro-volcanic conditions, the thermite melt reacts with water-saturated quartzo-feldspathic sand producing experimental spherules^14,15^. It is also known that submarine hydrothermal vents emitting hot fluids rich in trace metals and Fe-rich particles may also emit spherical-shaped particles^14,16,17^. Moreover, studies have shown that sediments influenced by hydrothermal activity consist of magnetite spherules, native Al spherules, and particles, Ti-rich particles, Mn-rich globules, Fe–Si rich metalliferous sediments^2,14,18^. In contrast, it is hard to find any literature which describes the hydrothermal-related origin of wustite-magnetite bearing spherules.

Marvin and Einaudi^4^ proposed that it is difficult to rule out the origin of Mn-bearing spherules from a cometary meteor since hardly much is known about the chemical composition of the comets and that Mn stars exist. However, based on morphological evidence and the absence of Ni, Finkelman^6^ suggested the volcanic origin of the Mn-bearing spherules. Also, it is known that magnetite, wustite, ferrihydrite, and palagonite phases in type-I cosmic spherules host Ni and do not consist of Mn^3^. It seems that magnetite and wustite-bearing spherules hosting Mn without any traces of Ni are considered non-cosmic in origin. However, the origin and formation conditions of such spherules are not well-constrained. In the present study, spherules extracted from the sediment samples from segment S2 of the CIR are analyzed to present a hypothesis explaining the genetic processes associated with their formation with implications to their origin.

## Results

**Figure 1:**
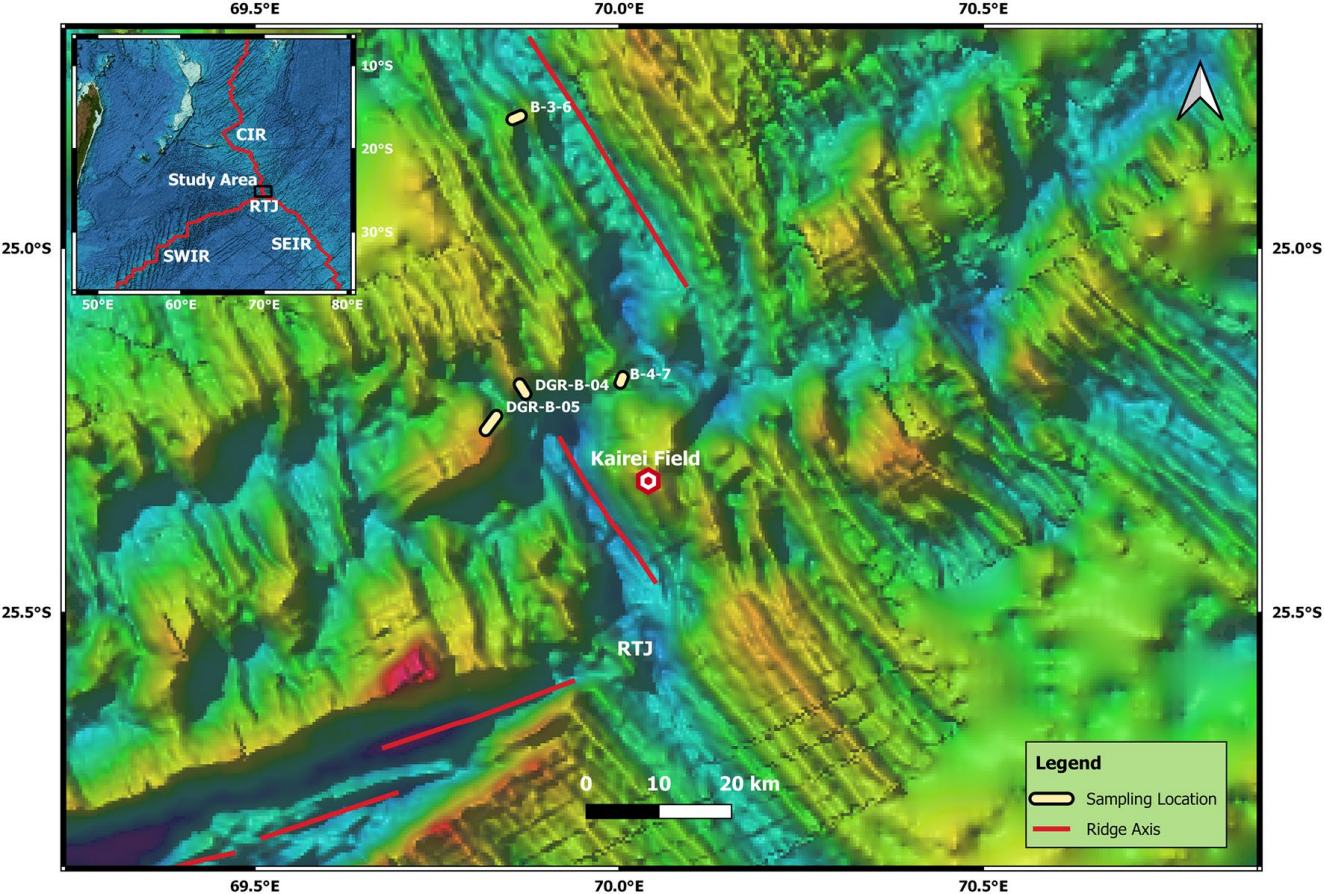
Bathymetry map of the study area showing sample location and known vent fields. Data is taken from the General Bathymetric Chart of the Oceans (GEBCO), 2019.

**Figure 2:**
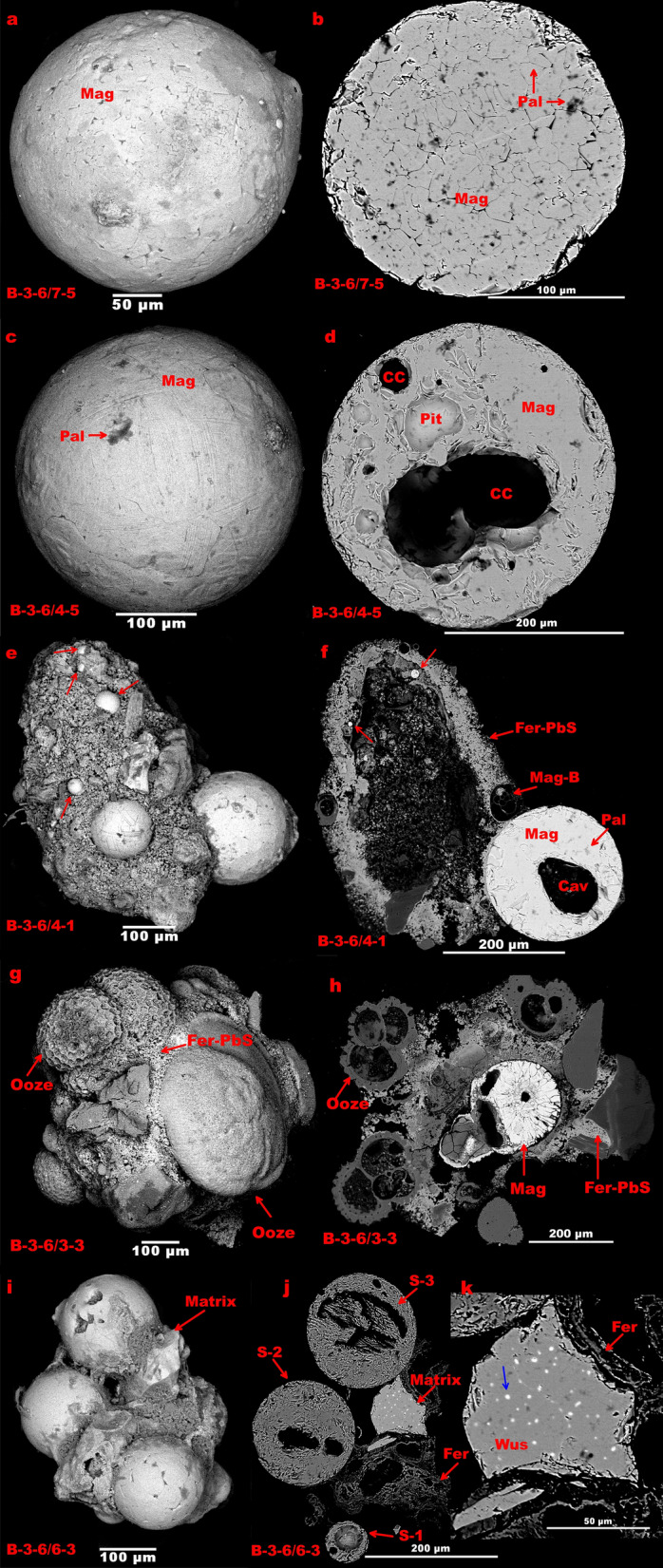
Spherules from the Central Indian Ridge. (**a**,**c**,**e**,**g**,**i**) Showing the external surface of the spherules. (**b**,**d**,**f**,**h**,**j**,**k**) Showing the polished section. Here, Wus—wustite, Mag—Magnetite, Pal—Palagonite, CC—Cylindrical cavity, Fer—Ferrihydrite.

A sediment sample with high abundance (19 spherules in ~ 85 g) of spherules is recovered from segment S2 of CIR (70° 54′ E, 25° 14′ S to 70° 50′ E, 24° 41′ S) (Fig. 1). The extracted spherules are spherical, ellipsoidal, black, and highly reflective and range in diameter from 250 to 500 µm in > 250 µm fraction. Externally, the surface appears polished with few surface imperfections, small depressions (pits or protrusions). Magnetite spherules have hexagonal/cubical mineral plates at the surface, which also have regular/irregular channels or mineral boundaries, sometimes with the radial arrangement, where the relatively poor reflectivity materials occupy the channels (Fig. 2a,b). Magnetite appears as crystals on the external surface, whereas wustite mostly appears as a homogenous glass phase (Fig. S1). Spherules also possess an inner cavity that is not always centrally located (Fig. 2c,d, S3). The inner surface of the cavity hosts features like interplate material exposer, pits, cracks, dendritic texture, and a collection of magnetite bubble-like features (Figs. S4, S6). Few spherules seem to be weathered post-deposition, and in a few cases, the weathered part is coated with carbonate precipitates (Fig. 2).

The composition of the spherules from the present study significantly vary from the well-studied type-I cosmic spherules, e.g., Genge et al.^3^, as the mineral assemblages magnetite (Fe_3_O_4_) and wustite (FeO) in them dominantly hosts a minor amount of Mn phase (Tables S2,S3). The predominant magnetite phase in the spherules has a heterogeneous composition of Fe (68.13–71.94 wt%) relative to wustite Fe (73.26–75.28 wt%). The wustite phase in the spherules consists of higher Mn (0.29–0.58 wt%) distributed heterogeneously relative to lower Mn (0.20–0.33 wt%) distributed homogenously in the magnetite phase. None of the spherules is found to contain the Ni phase, a characteristic of micrometeorites, demonstrating that the spherules recovered from CIR are non-cosmic in origin.

The concentration of major elements, Al, Si, P, Ca, Na, Mg, and Mn, show a large variation in the magnetite phase with undetectable K and Ti (Table S2). Similarly, the wustite phase also shows a large variation in major elements concentration, with Na, K, and Ti below the detection level (Table S3). The concentrations of Al, P, and Mg are comparable in both the magnetite (Avg Al = 0.15 wt%; P = 0.11 wt%; Mg = 0.11 wt%) and wustite phases (Avg Al = 0.14 wt%; P = 0.13 wt%; Mg = 0.10 wt%). The concentrations of Si, Ca, and Na are relatively higher in the magnetite phase (Avg Si = 0.30 wt%; Ca = 0.15 wt%; Na = 0.49 wt%) when compared to wustite phase (Avg Si = 0.20 wt%; Ca and Na are below detection limit). The trace metals V, Ni, Zn, and Cu, are not detected from any magnetite or wustite phases. However, minor Co is detected without much variation from most of the spherules in both magnetite (Avg Co = 0.02 wt%) and wustite (Avg Co = 0.02 wt%) phases. Besides, Cr and Pb are detected in the magnetite phase of spherule, B-3-6/4-5, and B-3-6/7-7, respectively, whereas the wustite phase showed no signals of Cr and Pb. The S (Avg = 0.30 wt%) is mainly present in the wustite phase. It is also observed that the S is mostly detected from the bright nano-particles present in the wustite phase, which may indicate the presence of sulfide nano-particles probably pyrrhotite. The low concentration of S from these nano-particles may represent matrix overlap and less representation of sulfide nano-particles owing to their small size.

Palagonite, a silica glass of basaltic composition, is present as a thin dark layer (< 2 µm) in between the mineral phase as irregular channels. Palagonite can be seen as a thin line where the palagonite is sectioned vertically or at an angle and as a thin film over the mineral phases where it is sectioned horizontally. The chemical analysis of these layers shows the presence of Fe as the primary phase and Si, Al, and Na in a minor amount, which may represent matrix overlap from the nearby Fe-mineral phases. Hydrous iron oxide replacing magnetite/wustite phases and occurring either within the particle or at the grain boundary have low analytical totals. Analyses of these hydrous phases show relatively low totals of 73–88 wt% due to water content, and are dominantly composed of Fe with a minor amount of Si (0.31–8.05 wt%), Al (0.06–0.24 wt%), Na (0.38–0.71 wt%), Mg (0.06–1.34 wt%), Ca (0.1–1.41 wt%), P (0.08–0.32 wt%), and Pb (0.37–1.20 wt%). The composition of these ferrihydrites (FeOOH) (Table S4) is distinct from the ferrihydrite observed in micrometeorites^3^. No other phases like chromite, metal bead, and PGE nuggets, which are generally present in cosmic type-I spherules, are present in the studied spherules from CIR (Genge et al.^3^ and references therein).

The spherules were analyzed using Confocal Laser Raman Microscope for identifying the minerals. We found that the magnetite showed absorption peaks near 660 Cm^−1^, 545 Cm^−1^, and 305 Cm^−1^; wustite showed absorption peaks near 650 Cm^−1^; pyrrhotite showed absorption peaks near 210, 275, 380, 525, 580, and 1280 Cm^−1^ (Figure S7).

## Discussions

### Constraints on the formation of spherules: Inferences from morphological features

The surface features of the CIR spherules do not display the characteristics of aerodynamic ablation products of the meteorites or spherules formed by melting and oxidation of cosmic particles during their flight through the atmosphere^3,11^, as they do not possess dendritic texture or metallic core. The magnetic particles derived from volcanic eruptions are generally ellipsoidal in shape as they evolve from the molten droplets^6,19^. The molten fluids emanating from the hydrothermal or magmatic activity may form spherical particles as they experience quenching and hydrostatic pressure in the water column. Most of the single un-fused spherules (Fig. 2a,c) from CIR show a thin outer layer either composed of smaller magnetite plates compared to the inner part or the presence of numerous fractured features, and this may indicate rapid cooling of the outermost part, forming a thin, rigid shell on the molten droplets. It seems that because of the significant difference in density and cooling rate between Fe and Si, the Si formed Si-rich glass micro-layers via fast cooling, and the Fe-rich liquid-cooled relatively at a slower rate forming euhedral magnetite crystals^14^. Conversely, the wustite spherules do not consist of such Si phases and crystals and are rather present as homogenous glass phases.

The inner part of the spherule remains at a relatively higher temperature than the outer part during the cooling process since the excess oxygen, which has good solubility in the molten Fe phase, gets entrapped in the inner part forming cavities^20^. Experimental studies have also shown that the percentage of hollow spherule and the cavity’s size directly depends on the oxygen fugacity^20^. Oxygen fugacity may also control the presence of magnetite and wustite phase, with magnetite forming at relatively higher oxygen fugacity^20^. Cosmic spherules may lose volatiles forming cavities by oxidative heating during their flight through the atmosphere, whereas non-cosmic spherules, especially those forming on the ocean floor, may experience volatile loss during the quenching of the molten droplets. Microbial activities may also form such cavities in the spherules. Otherwise, such cavities can form in cosmic spherules if the metal (Fe, Ni) cores have been rinsed. However, cavities together with mineral chemistry results, i.e., the presence of Mn and the absence of Ni, demonstrate the non-cosmic origin of the CIR spherules.

### Genetic constraints on the origin of spherules: Mn hypothesis

Finkelman^6^ classified the spherules of extraterrestrial origin into two groups, one originating from the ablation of iron meteorites and the other from the stony meteorites. Both the extraterrestrial origin groups host a significant amount of Ni in both the wustite and magnetite phases, and however, he classified the spherules hosting Mn as non-cosmic in origin. It is also challenging to define a fractionation process where Mn (0.9) relative to Fe (100) would be retained, removing all other elements with higher abundance in the solar system, like Cr (1.3), Ni (5.2)^4,21^. Moreover, since the evaporation point of Mn (2061 °C) is lower than that of Cr (2672 °C), Ni (2730 °C), and Co (2927 °C), it becomes difficult to establish how an element with a lower boiling point is retained, and an element with higher boiling point has escaped, if the spherule is to be of cosmic origin and has experienced high temperature melting (> 2000°C^22^) and oxidation during atmospheric entry.

Recovery of magnetite spherules hosting Mn phase from different geological environments including the Pleistocene sand, Greenland ice caps^4^, 16 million-year-old ferromanganese crust from the Mid-Atlantic Ridge^23^, several million-year-old manganese oxide crusts and manganese nodules^6^, and CIR sediments reported in the present study, rules out the possibility of them being contaminated by industrial processes^24^. Also, it is not easy to distribute the industrial spherules, specifically of bigger size (> 250 µm), to the study location at CIR, which is ~ 1000 km from the nearest land source.

**Figure 3:**
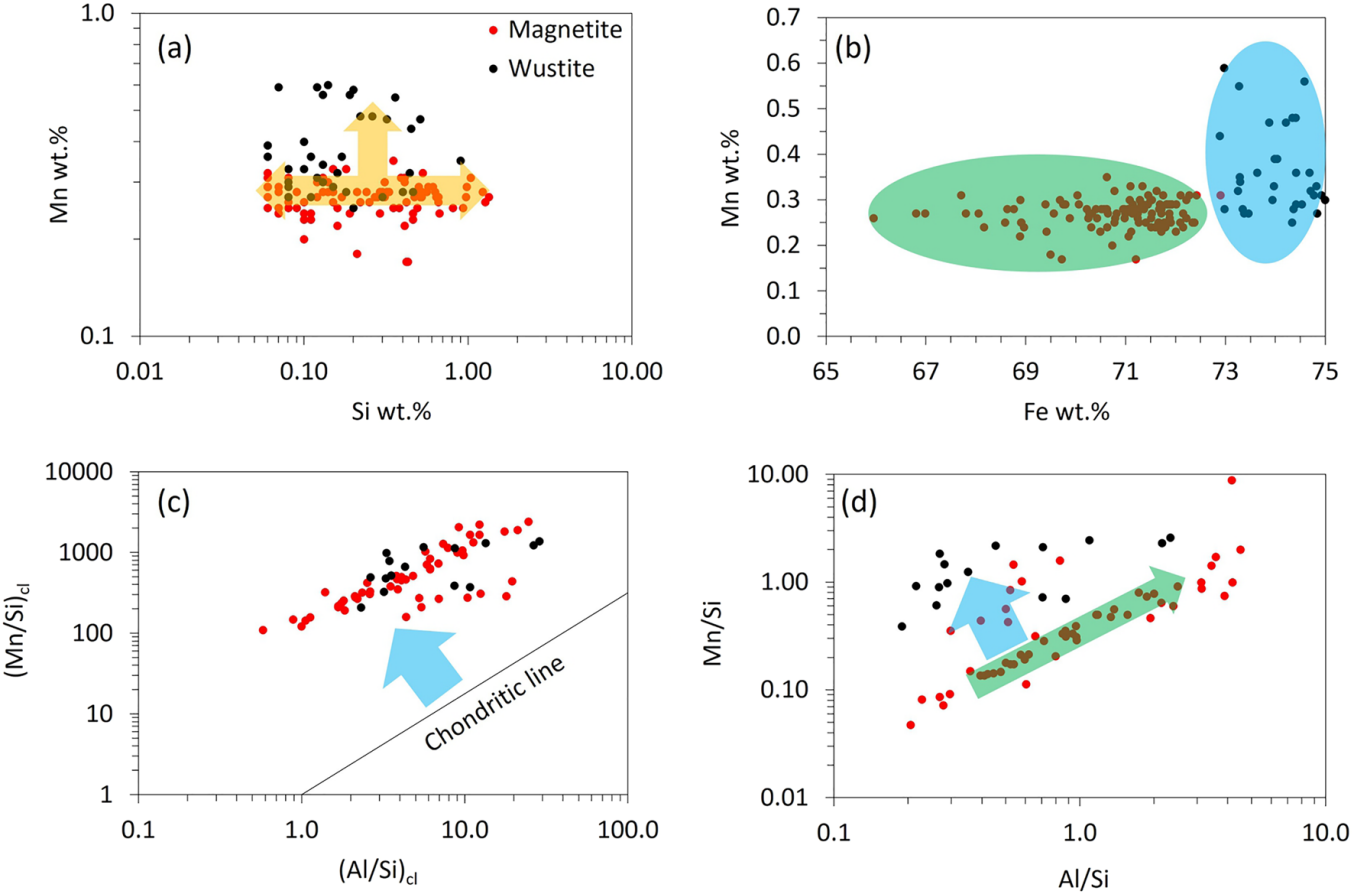
(**a**) Si versus Mn wt%, (**b**) Fe versus Mn wt%, (**c**) Al/Si_cl_ versus Mn/Si_cl_, and (**d**) Al/Si versus Mn/Si, shows the magnetite (red) and wustite (black) plots.

The distribution of Mn in Fe-oxide is independent of the Si phase (Fig. 3a). Fe has higher oxidation potential compared to Mn, and Mn oxidation is kinetically limited at lower temperatures^25^. Magnetite forms at relatively higher oxygen fugacity than that of wustite, indicating that at higher oxygen fugacity, Mn is more homogeneously distributed in the Fe-oxide phase (Fig. 3b). However, higher Mn concentration in the wustite than magnetite may indicate that Mn association in the Fe-oxide phase is less dependent on the oxygen fugacity of the system. Furthermore, earlier studies on wustite have shown that MnO forms a solid solution with wustite and can stabilize the crystal structure of wustite^26^.

In the chondrite normalized plot (Fig. 3c), the magnetite and wustite are not fractionated with respect to each other but are fractionated from the chondritic composition incorporating higher Mn. On the contrary, the Al/Si vs. Mn/Si plot (Fig. 3d) showing the un-normalized values indicates that the wustite phase is fractionated from the magnetite phase incorporating higher Mn. This further indicates the same source for magnetite and wustite in the spherules, most probably mantle in this case. Later, when molten droplets/hydrothermal fluids fractionated from the source "mantle composition," forming magnetite and wustite in the spherules, the wustite differentiated from magnetite incorporating higher Mn (Fig. 3d). The above observation indicates that the spherules hosting magnetite and wustite phases may have originated by the interaction of reduced hydrothermal fluids with the ultramafic/basaltic rocks or silica-undersaturated magmatic melts. However, this is not the only way to form such spherules, other processes that can form such spherules are described in the following paragraph.

Alteration of cosmic spherules via seawater and diagenesis may lead to Mn enrichment or removal of Ni from the wustite and magnetite phase. However, this principle lacks any proven evidence through scientific studies on spherules, as per our knowledge. Also, the discovery of cosmic spherules from Myanmar jadeitite, a rock type from subduction zone forming at high pressure (> 1 GPa) and relatively low temperatures of about ~ 250–370 °C, indicates that the cosmic spherules can withstand such alteration conditions^7^. Though the spherules from Myanmar jadeitite consist of higher Mn (up to 2 wt%) in the cortex, they also consist of Ni and pure iron cores^7^. Nevertheless, the CIR spherules do not host detectible Ni and pure iron cores. Further, low-temperature alteration processes leading to the incorporation of Mn in the wustite contradict the oxidation kinetics of Fe and Mn. In addition, the relatively higher Mn concentration in the wustite compared to magnetite further contradicts the low-temperature oxidation kinetics of Fe and Mn^25^. However, alteration of original particles via Mn-bearing hydrothermal fluids, such as released during the serpentinization of ultramafic rocks, is one possibility for the formation of CIR spherules. In such a case, it is difficult to confirm the original particle, which can be cosmic spherules, volcanic spherules, or even foraminifera. Altogether, the detailed mechanisms for the formation of CIR spherules are still doubtful, but it seems that these spherules are significantly influenced by hydrothermal fluids.

### Wustite: constrains on its formation

Under high reducing conditions, i.e., at very low oxygen fugacity, Fe^3+^ in magnetite reduces to Fe^2+^ and forms wustite, and the further reduction of wustite may form native iron. The equilibrium assemblage of wustite-magnetite is commonly found in micrometeorites of extraterrestrial origin and is rarely found in the terrestrial environment because of its selective formation under low-oxygen-fugacity, high-temperature, and silica-undersaturated environments^26^. The rare occurrence of wustite from the terrestrial environment includes, associated with Ni-sulphide deposits; carbonate-rich skarns and related hydrothermal veins; in association with magnetite and siderite in thermally metamorphosed coal; and as inclusions in diamond^3,26–28^.

At atmospheric pressures, wustite is unstable at temperatures below 570 °C and decomposes to magnetite and α-Fe; however, if formed may endure in a metastable phase^26^. Reduction of hematite below 560 °C and decomposition of CO_2_ to C may also form metastable wustite^26^. Besides, wustite may also form at low oxygen fugacity conditions (< 8.5 × 10^–31^)^26^. The Gold hunter hydrothermal vein of the Lucky Friday mine hosts rare wustite-magnetite assemblage along with galena, pyrite, and siderite. Interestingly, the wustite-magnetite assemblage from the Gold hunter hydrothermal vein consists of a minor amount of Mn, similar to the wustite-magnetite in spherules from CIR. Also, the Mn content in wustite is higher compared to that of magnetite, which is also the case for the CIR spherules. Since the wustite from CIR spherules is similar in composition to that of wustite from the hydrothermal vein of the Lucky Friday mine^26^, it may indicate that hydrothermal fluid forming wustite in CIR spherules formed below 363 ± 10 °C. Interestingly, the wustite from the Ni-sulphide deposit of Disko Island, formed by the reduction of basaltic melts by carbon, contains no detectable Ni^27^. The observation that, absence of Ni, a characteristic element of extraterrestrial spherules, and the presence of Mn, a characteristic of hydrothermal wustite, suggests a hydrothermal origin for the spherules recovered from the CIR.

### Pyrrhotite: constrains on its formation

Pyrrhotite can form from magmatic activity, bacteriogenic reduction in the sedimentary environment; metamorphic activity forming meta-sediment; and hydrothermal activity^29^. Pyrrhotite or troilite (stoichiometric FeS) can be found in unaltered basalt, mantle xenoliths, diamonds, meteorite, lunar basalts, and probably in Earth's mantle and core^29^. Pyrrhotite may also form rounded inclusions in igneous rocks because of the immiscibility of the sulfide liquid in the silicate magma^29,30^. The simplified redox reaction after Hall^29^ forming pyrrhotite is as follows:

1 $${\text{2FeS }} \leftrightarrow {\text{ FeS}}_{{2}} + {\text{ Fe}}^{{{2} + }} + {\text{ 2e}}^{ - }$$

Pyrrhotite is a prevalent hydrothermal precipitate from reduced hydrothermal fluids forming syn-sedimentary mineralization^29,31^. Since pyrrhotite's solubility is more than other common metal mono-sulfides, it is considered a dissolved phase in most hydrothermal solutions^29^. Haymon^32^ has suggested the precipitation of pyrrhotite at a hydrothermal site from the East Pacific Rise; however, here, the pyrrhotite is rapidly oxidized, forming limonite, pyrite, and Cu-Fe sulfides. The progressive cooling of hydrothermal fluids leads to a relative increase in *f*_O2_ and *f*_S2_, which signifies that pyrrhotite stabilization occurs at relatively higher temperatures when compared with pyrite stabilizing at a lower temperature^29,33^.

A fugacity-fugacity diagram of Fe–S–O phases at 340 °C from D'Amore and Gianelli^33^ is used to interpret the formation condition of pyrrhotite observed in the CIR spherules. Pyrite can exist at relatively higher oxygen and sulfur fugacity compared to pyrrhotite. The sulfide nano-particles present as disseminations in the spherule B-3-6/5-8 and 6-3 (Fig. S5 and 2k), hosted by wustite are pyrrhotite, as wustite shares the phase boundary with pyrrhotite and not with pyrite (see Fig. 7 of D'Amore and Gianelli^33^). So during the formation of these Fe-oxide phases, the oxygen and sulfur fugacity might be below the triple junction point connecting FeO–Fe_2_O_3_–FeS (see Fig. 7 of D'Amore and Gianelli^33^). In particle B-3-6/6-2, two sulfide nano-particles can be seen (Fig. S5f), the one present at the top-left corner is embedded in the magnetite phase, and the other present at the bottom-right corner occurs at the phase boundary between wustite and magnetite. The presence of sulfide nano-particle and magnetite together with wustite phase signifies the sulfide nano-particle to be pyrrhotite, and the spherule B-3-6/6-2 might have formed at *f*_O2_ (~ 10^–37^) and *f*_S2_ (~ 10^–18^) (see Fig. 7 of D'Amore and Gianelli^33^). Also, the presence of sulfides as nano-particles indicates very low *f*_S2_ in the system, which leads to the formation of disseminated pyrrhotite rather than pyrite. Further, under higher alkalinity and lower *f*_S2_ conditions with constant *f*_O2_, pyrrhotite and magnetite phase may co-precipitate along with high sulfur fluids^29^.

The Gold hunter hydrothermal vein of the Lucky Friday mine hosts Ag–Pb–Zn replacement veins along with wustite-magnetite assemblages^26^. Similarly, the presence of magnetite spherules and a minor quantity of Pb and S in the ferrihydrite matrix (Fig. 2f,h) is a direct signature for the hydrothermal origin of the CIR spherules, reported in the present study. Also, the bulk geochemical composition of the sediments shows the presence of ~ 300 ppm of Pb, which is much higher than the Pb (~ 50 ppm) in the background sediments^34^.

## Conclusions

Not much literature is available that reports the hydrothermal-related origin of the magnetite-bearing spherules, and hardly any literature discusses the hydrothermal-related origin of wustite-magnetite-bearing spherules. This study is the first report describing the recovery and characterization of hydrothermal-origin spherical-shaped particles. Instead of different morphological features, all these spherules have a resemblance, where all of them host Mn in the magnetite and the wustite phase and are devoid of Ni. The absence of Ni and the presence of Mn suggests the spherules to be non-cosmic in origin. The presence of Mn in the wustite phase further indicates the spherules to be of hydrothermal origin.

Relatively higher concentration and heterogeneous distribution of Mn in wustite compared to lower concentration and homogenous Mn in magnetite indicate that they formed under low oxygen fugacity conditions similar to hydrothermal vents. Apart from Mn, the presence of sulfide nano-particles in the wustite phase and a minor quantity of Pb and S in the ferrihydrite matrix indicates that CIR spherules are of hydrothermal origin.

It is envisaged that the CIR spherules, hosting magnetite and wustite phases originated either by the interaction of the reduced hydrothermal fluids with the ultramafic/basaltic rocks or silica-undersaturated magmatic melts, or by alteration of original particles (such as cosmic spherules, volcanic spherules, or even foraminifera) via Mn-bearing hydrothermal fluids, such as released during the serpentinization of ultramafic rocks.

A high abundance of bigger spherules (250–500 µm), 19 spherules in ~ 85 g of the sediment sample B-3-6 at ~ 85 km north of the RTJ, indicates the presence of hydrothermal activity in the near vicinity.

## Methods

The study area falls in the segment S2 of CIR with respect to RTJ between 70° 54′ E, 25° 14′ S and 70° 50′ E, 24° 41′ S spanning a length of ~ 65 km (Fig. 1). The studied sediment samples are collected from the study region using a cylindrical pipe of ~ 15 cm diameter and ~ 1 m in length attached to a dredge (Fig. 1, Table S1) during the scientific cruises onboard R/V Akademik Nikolaj Strakhov (ANS- leg 4) and R/V Sagar Kanya (SK-300). The samples are collected precisely from four principal locations: the first location (B-3-16) is in the northern part of segment S2 and lies west of the neo-volcanic mount (Table S1, Fig. 1). The other three locations are close to the transform fault between segments S1 and S2. B-4-7 location lies in the transition zone of the two segments, and B-05 location lies in the northeastern flank of the 25° S OCC (oceanic core complex)^35^. Sample B-04 is collected from the termination point of the slope of an older axial ridge, terminating at the transform fault between segments S1 and S2. A detailed bathymetry map of the regions is analyzed before sample collection, and the regions with topographic lows in and around the significant mounts are selected explicitly for sampling. The studied sediments are distinguished based on their appearance, which is dark to pale brown in appearance, and the sediments are mostly coarse-grained (Table S1). The majority of the sediment samples consist of pelagic sediments with calcareous fossils and foraminiferal oozes.

All four sediment samples are taken for identifying potential samples with magnetic particle enrichment. Samples are first washed for the removal of superfluous matter using distilled water (3 times). Then sediment samples were wet sieved using 60 mesh (250 microns) sieve and dried overnight in an oven at 60 °C. Samples with > 250-micron fraction are taken for hand-magnet separation for separating magnetic grains from non-magnetic grains. These magnetic grains were then taken under a binocular microscope to pick potential hydrothermally derived Fe-rich components and spherical grains. Sediment sample B-3-6 having higher spherule density (Table S1) in the > 250 µ fraction is taken for component-level analysis, i.e., back-scattered imaging of whole-grain and polished grains, and EPMA spot analysis.

Back-scattered electron imaging of the handpicked magnetic grains is conducted on a JEOL-JSM 6360LV Scanning Electron Microscope (SEM) at the National Center for Polar and Ocean Research (NCPOR), Goa. The grains, including spherules, are mounted on double-sided carbon tape attached to 1 cm diameter aluminum stubs and sputter-coated with carbon. Mounted minerals are taken for back-scattered electron imaging (BSEI) of the whole grain. An accelerating voltage of ~ 15 kV and a beam current of ~ 4 nA are used with a focused beam.

Following whole-grain imaging, extracted spherules from sample B-3-6 are taken for BSE imaging of the polished section and quantitative spot analysis using EPMA. Magnetic particles, including spherules mounted on aluminum stubs from sample B-3-6, are mounted on epoxy resin, grinded, polished and carbon-coated. In total, ~ 19 magnetic particles, including spherules, are then taken for back-scattered electron imaging (BSEI) and spot analysis on an SX five electron probe microanalyzer using wavelength-dispersive spectroscopy (EPMA-WDS) installed at NCPOR-Goa. All the spherules are imaged at an accelerating voltage of 15 kV, beam current 4nA, and a working distance of 15 mm. A focused beam is used for the elemental analysis of the spherules (Major elements: Al, Si, Ca, Fe, Mg, Mn; and minor elements: S, Ti, Cr, V, Co, Ni, Zn, Cr, Pb, and Cu). A defocused beam of 10 µm diameter is used for analyzing sensitive elements (Na, K, and P). In addition, sensitive elements Na and K are analyzed in an integrated approach of sub-counting using four steps of 5 s. The counting time is 20 s for the peak and 10 s for the background. The overlap corrections of Ti, Cr, V, Fe, and Zn are employed on V, Mn, Cr, Co, and Na, respectively. All the spherules are analyzed at an accelerating voltage of 15 kV, beam current 20nA, and a working distance of 15 mm. For calibration of the instrument following standards are used, Na—Albite, Mg—Peridot, K—Orthoclase, Ca—Diopside, Ti—TiO_2_, Fe &S—Pyrite, V—V-metal, Cr—Cr_2_O_3_, Pb—Pyromorphite, Al—Al_2_O_3_, Si—Wollastonite, P—Apatite, Co—Co-metal, Ni—Ni-metal, Zn—ZnS, Mn—Rhodonite, Cu—Cu-metal. Several spots are analyzed on each spherule for gathering better information on the spherules. The detection limit (DL) and standard deviation (STD) for all the elements analyzed are below 500 ppm and ± 200 ppm, and data points of any element of any particular analysis having values greater than DL & 3*STD of the respective element analyzed is only considered.

The analytical totals and stoichiometry of Fe-oxide are examined to evaluate the quality of WDS analyses. The analysis of small phases or phases proximal to cavities and grain boundaries showed matrix overlap from different materials or analytical total < 100%. The ideal analytical total of magnetite based on oxygen bound to Fe^2+^ is 93 wt%^3^, and when stoichiometrically converted to Fe_3_O_4_ (magnetite), will give a total of ~ 100 wt%. The analytical total of 93 ± 3 wt% is assumed to be accurate for magnetite compositions. However, the Fe–O phase relation in wustite may result in stoichiometry with excess oxygen of 1.0–4.1 atomic% (0.7 2.9 wt%) because of vacancies^3,36^. Analytical totals between ~ 96 and ~ 100 wt% are considered ideal for wustite. The spots where the analytical total is < 90% are considered less representation of the total owing to overlapping cavities or grain boundaries. However, proper interpretation is drawn for other altered Fe-phases with < 90% total together with morphological features.

## Geological setting

CIR, whose rift valley trends towards NNW of RTJ (25° 30′ S) is categorized as a slow-spreading ridge with an average full opening rate of ~ 47.5 mm/year^37^. The CIR axis is marked by an axial valley of 5–35 km wide and 500–1800 m deep, having neo-volcanic ridges ranging in height from 100 to 600 m at some places^38^. An oceanic core complex (OCC) lies ~ 15 km east of the Kairei Hydrothermal Field (KHF)^35^. The southern part of the CIR is marked by the presence of one inactive and two active hydrothermal vent fields, namely, MESO, Edmond, and Kairei at 23° 23.56′ S, 69° 14.53′ E; 23° 52.68′ S, 69° 35.80′ E; and 25° 19.23′ S, 70° 02.42′ E, respectively^39,40^. The study area falls in segment S2 of CIR with respect to RTJ, where the Axial valley depth ranges from 3600 to 4200 m with Axial valley width of 25 km and consists of a significant neo-volcanic ridge with a height of ~ 300m^38^.

## Supplementary Information

Supplementary Information.
