# Supplementary material for: Recovery of hydrothermal wustite-magnetite spherules from the Central Indian Ridge, Indian Ocean
Source: Sci Rep. 2022 Apr 26;12:6811. doi: 10.1038/s41598-022-10756-1 (PMC9042910; doi:10.1038/s41598-022-10756-1)
Supplement: Supplementary file 1 — Supplementary Information. [file 41598_2022_10756_MOESM1_ESM.docx]

**Supplementary Material to**

**Recovery of hydrothermal wustite-magnetite spherules from the Central Indian Ridge, Indian Ocean.**

Deepak K. Agarwal, Palayil John Kurian

National Centre for Polar and Ocean Research, Ministry of Earth Sciences, Vasco-da-Gama, Goa – 403804, India

1. **Morphology and texture of spherules**
   1. *Magnetite spherules*

Magnetite spherules (B-3-6/6-7, 7-5, and 4-6) with compact structures have a high degree of sphericity (Fig. S1a, c, e). The surface of the spherules shows an interlocking plate structure. The plates showing radial/interlocking arrangement at the surface are either partly or completely fused together. Also, these plates are separated by distinct gaps or fissures, which are either empty or are filled by matter that protrudes like mortar between bricks. FigureS1b, d, f shows the polished section of the spherule B-3-6/6-7, 7-5, and 4-6, respectively. Here, the internal structure of the spherule indicates the presence of subhedral to anhedral magnetite minerals. Palagonite is present as a thin dark layer in-between the mineral phases of magnetite, and this can be seen as a thin line where the palagonite is sectioned vertically or at an angle and as a thin film over the mineral phases where it is sectioned horizontally. The spherule B-3-6/6-7 and 4-6 also have a small broken area (crater-like indent) where the surface minerals are not present. Mineral plates at the edge of the broken part of the spherule B-3-6/6-7 collapse inward. Spherule B-3-6/7-5 is formed of two distinct platy magnetite minerals with different sizes as seen on the external surface, and the area with relatively smaller magnetite minerals is darker in appearance. Spherule B-3-6/7-5 also shows the presence of several small bulges. The internal structure of the spherule B-3-6/7-5 also indicates that the spherule is composed of magnetite minerals of different sizes (Fig. S1d). The external surface of the spherule B-3-6/4-6 at the top-right portion has a smooth surface (Fig. S1e), and the same portion, when seen in the polished section, is brighter and is composed of wustite (Fig. S1f).

- 1. *Magnetite spherule hosting cylindrical cavity*

Magnetite spherules hosting cylindrical cavity have deformed except spherule B-3-6/4-5, which has a high degree of sphericity (Fig. S2). The curvature of the deformed spherules has decreased, and thus they diverge from spheres. SpherulesB-3-6/4-5 and 5-5 are composed of homogenous magnetite glass, whereas spherules B-3-6/7-6 and 5-6are formed of magnetite mineral plates. In B-3-6/5-6, magnetite minerals can only be seen on the external surface and are 30-40 µm in size (Fig. S2e). On the contrary, spherule B-3-6/7-6 have smaller platy minerals, visible only in the central part of the external surface, and the internal structure reveals that it is composed of subhedral/anhedral magnetite minerals (Fig. S2c, d). Also, in spherule B-3-6/7-6, the number of magnetite minerals increases and gets smaller towards the periphery of the spherule and cylindrical cavity (Fig. S2d). The polished section of all these spherules shows crack-like features throughout the perimeter of the spherules and cavities. The spherule B-3-6/5-6 hosting one central cavity and B-3-6/6-6 hosting multiple overlapping cavities are primarily hollow; as a result, they are similar to a magnetite shell (Fig. S2f, h). Spherule B-3-6/4-5 (Fig. S2a) has parallel stripes all along the surface, a dark spot (probably palagonite), and a slight bulge; otherwise, the external surface appears to be smooth. The polished section of the spherule B-3-6/4-5also shows the presence of two overlapping cylindrical cavities (CC) at the center, a smaller cylindrical cavity towards the top-left edge, and pits or vesicles (Fig. S2b). B-3-6/7-6 consists of two cylindrical cavities (CC) and a subhedral cavity with ferrihydrite (Fer) at the periphery. B-3-6/7-6 also has a depression with several bulges surrounding it towards the lower portion (Fig. S2c). The spherule B-3-6/6-6 also shows the presence of porous overgrowth and external carbonate precipitates (Fig. S2g).

- 1. *Spherules composed of wustite and magnetite*

Spherules composed of wustite and magnetite have a low degree of sphericity and seem to be weathered and coated with external seawater precipitates post-deposition except for B-3-6/4-4 (Fig. S4). Spherule B-3-6/4-4 has rough morphological features at the surface and has few dark spots (Fig. S4a). The polished section of the spherule B-3-6/4-4 displaysmultiple overlapping cylindrical cavities (CC) forming a big cavity and a separate smaller cylindrical cavity (Fig. S4b). Magnetite is mainly present in-between the wustite minerals. Also, a magnetite rim (MagR) can beobserved around the spherule. In the magnified image of B-3-6/4-4 (Fig. S4c), magnetite (Mag), wustite (Wus), and pit can be seen, and the pit hosts magnetite bubble (MB) and magnetite crystals (MC). The spherule B-3-6/7-7 has a hollow interior with a smooth surface, the shell is composed of magnetite (Mag) and wustite (Wus) glass, with no proper minerals visible, and magnetite mainly occupies the outer rim of the spherule (Fig. S4d, e, f).B-3-6/6-5 is a twin spherule, where the bigger spherule appears to have platy minerals at the surface, whereas the smaller spherule has a smooth surface (Fig. S4g). In the polished section, only the bigger spherule is exposed and is composed of magnetite (Mag) and wustite (Wus) shell, magnetite mostly occupying the outer rim, and the sizable cavity seems to be composed of multiple overlapping smaller cavities (Fig. S4h, i). In B-3-6/5-7, three smaller spherical spherules are fuse to a bigger ellipsoidal spherule, the bigger spherule seems weathered, and no mineral plates are visible, whereas the smaller spherule at the top is composed of euhedral minerals (Fig. S4j). The polished section of B-3-6/5-7 shows that the bigger spherule is composed of a homogenous wustite phase, hosts a cylindrical cavity, circular ferrihydrite, weathered ferrihydrite, and cracks at the periphery of spherule and cavities (Fig. S4k, l). The exposed smaller spherule is formed of magnetite phase and seems not a fused but a pop-out structure with a small wustite neck; it also consists of cracks throughout the spherule (Fig. S4l).

- 1. *Spherules hosting sulfidenano-particle*

Spherules hosting sulfidenano-particlesareprimarily composed of wustite and host the sulfide particleswithin the wustite phase (Fig. S5). However, B-3-6/6-2 seems to host two sulfide particles in the magnetite phase (Fig. S5f). All thesespheruleshave a low degree of sphericity and appear weathered. B-3-6/5-8 spherule is bulbous and has rough morphological features at the surface, which appear to be a porous overgrowth, and are seen all over the spherule (Fig. S5a). The polished section of B-3-6/5-8 shows that it is formed of homogenous wustite phase with numerous micron to sub-micron size sulfide particles (pointed by the blue arrow) and consists of ferrihydrite phases (Fig. S5b, c). The bigger sulfide particles, around ~ 1-3 µm in size, are present at the right-hand side periphery,whereas sub-micron-sizedsulfide particles havescattered in the wustite phase. B-3-6/5-8 also hosts a sizable cavity surrounded by ferrihydrite and smaller weathered cavities. In B-3-6/6-2, multiple wustite spherules are fuse to the lower-left of a bigger and broken spherical grain (Fig. S5d). The polished section shows that B-3-6/6-2 is mainly composed of a homogenous wustite phase with a magnetite layer present at the upper part of the section and a magnetite rim (MagR) present on the lower left (Fig. S5e). Numerous micron size magnetite phases are also present at the central part of the spherule along with two sub-micron-sizesulfide particles (Fig. S5f). In particle B-3-6/6-3, three magnetite spherules with a high degree of sphericity are fuse together by a Fe-oxide matrix (Fig. S5g). The polished section shows that B-3-6/6-3 particle also consists of wustite and ferrihydrite matrix apart from three magnetite spherules (Fig. S5h). The wustite phase consists of micron to sub-micron-size sulfide particles (shown by the blue arrow) of diverse size (Fig. S5i). Magnetite spherules consist of cylindrical cavities (CC) and crack-like features throughout the spherule.

- 1. *Spherules fused to sediments and ooze*

In particles B-3-6/4-1 and 7-4, multiple magnetite spherules with a high degree of sphericity are fused to the Fe-oxide matrix, consisting of Pb and S (Fig. S6a, c). On the contrary, in particles B-3-6/3-3 and 6-4, magnetite and wustite spherules, respectively, are fused with oozes, probably foraminifera (Fig. S6e, g). In particle B-3-6/4-1, the two bigger spherules have different surface features, parallel stripes, and platy minerals, respectively (Fig. S6a). The polished section shows that the bigger spherule is composed of a homogenous magnetite phase (Fig. S6b). The matrix is composed of ferrihydrite with a minor amount of PbS; also, two smaller magnetite spherules (pointed by red arrows) can be seen (Fig. S6b, Table4). The bigger spherule consists of an oval-shaped cavity (Cav)and palagonite (Pal), and above this spherule magnetite bubble (Mag-B) like features can be seen. In particle B-3-6/7-4 (Fig. S6c), the external surface of the spherules is smooth.A part of the matrix, having a dendritic pattern (DM-S), shows linear structures and consists of a significant amount of Cl and S. The polished section shows that the bigger spherule is hollow, and the interior features indicate that it is a magnetite shell made-up of hexagonal/cubical minerals arranged in a leaf/dendritic structure (Fig. S6d). A smaller magnetite spherule present over the bigger magnetite spherule also shows similar features. The matrix is composed of ferrihydrite with a minor amount of PbS (Fig. S6d, Table 1). In particle B-3-6/3-3, multiple oozes are fuse to ferrihydrite with a magnetite spherule located at the center (Fig. S6e, f). The spherule is composed of elongated magnetite minerals; also, it hosts a cylindrical cavity surrounded by relatively smaller magnetite minerals, unlike other spherules. The ferrihydrite matrix surrounding the magnetite spherule consistsof a minor amount of PbS. The Fe-rich precipitates in between the oozes also consist of PbS, and the foraminiferal oozes are fuse towards the outer periphery of the grain. In particle B-3-6/6-4 (Fig. S6g, h), wustite Spherule is fused to a foraminiferal ooze, where the fused part is composed of ferrihydrite. However, in the polished section, only wustite spherule is exposed, and it consists of several cylindrical cavities, magnetite-rim, and sub-micron-sizeeuhedral magnetite phases towards the magnetite rim.

1. **Raman Microscopy of the Spherules**
   1. *Methods*

The sample was re-polished before Raman spectroscopy. Raman spectra were collected using a Witec alpha 300 Confocal Laser Raman Microscope at IIT-Bombay, with a 532 nm green Ar^+^ laser source, focused through a 20x and 50× objective lens. The laser power used was 6 mWwith acquisition times of 50 s.

**
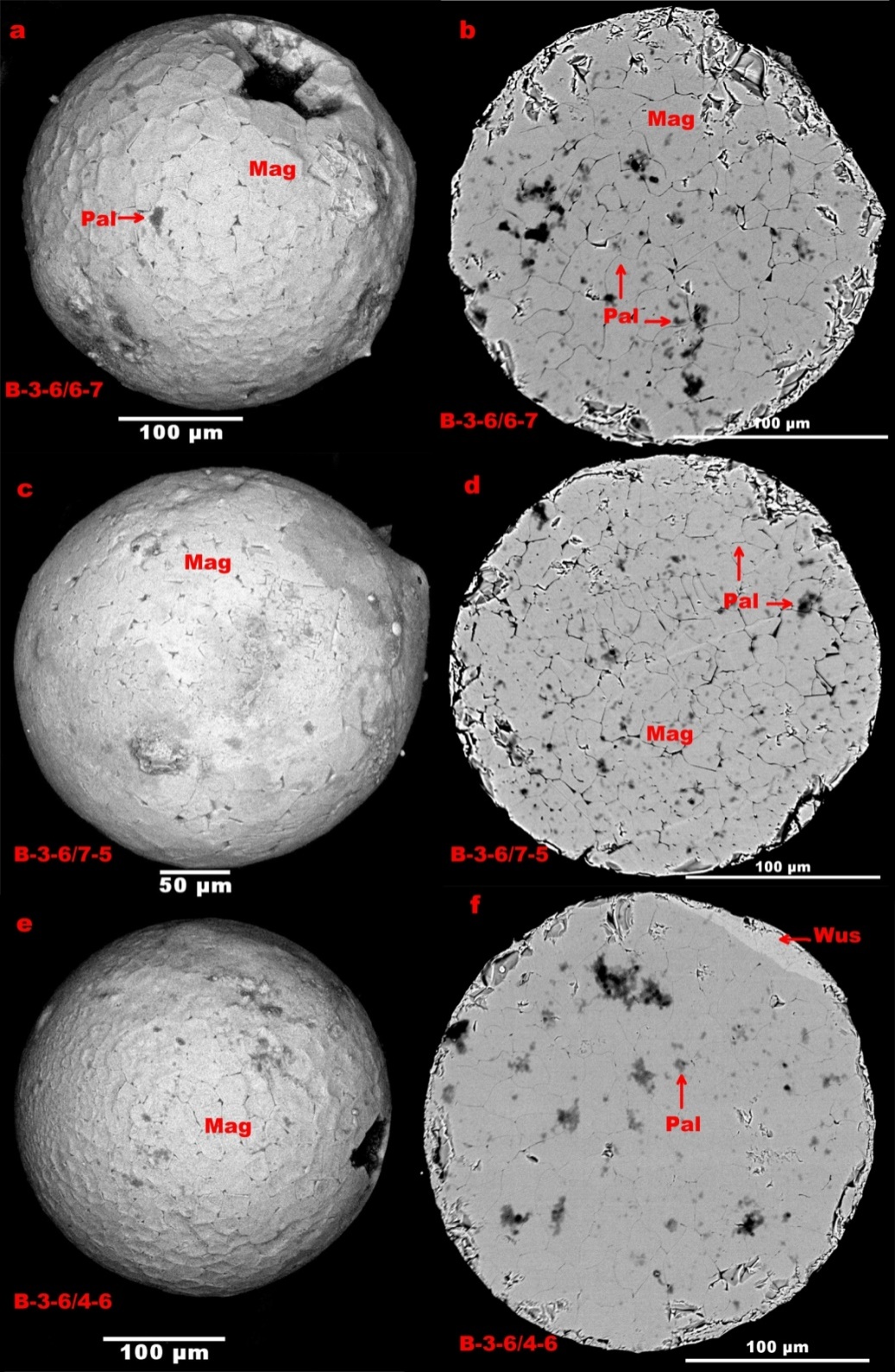
**

**Figure S1:** Magnetite spherules with compact structure. (a), (c), and (e) showing the external surface of the spherules B-3-6/6-7, 7-5, and 4-6, respectively. (b), (d), and (f) showing the polished section of the spherule B-3-6/6-7, 7-5, and 4-6, respectively. Here, Mag – Magnetite, Pal – Palagonite, Wus – wustite.


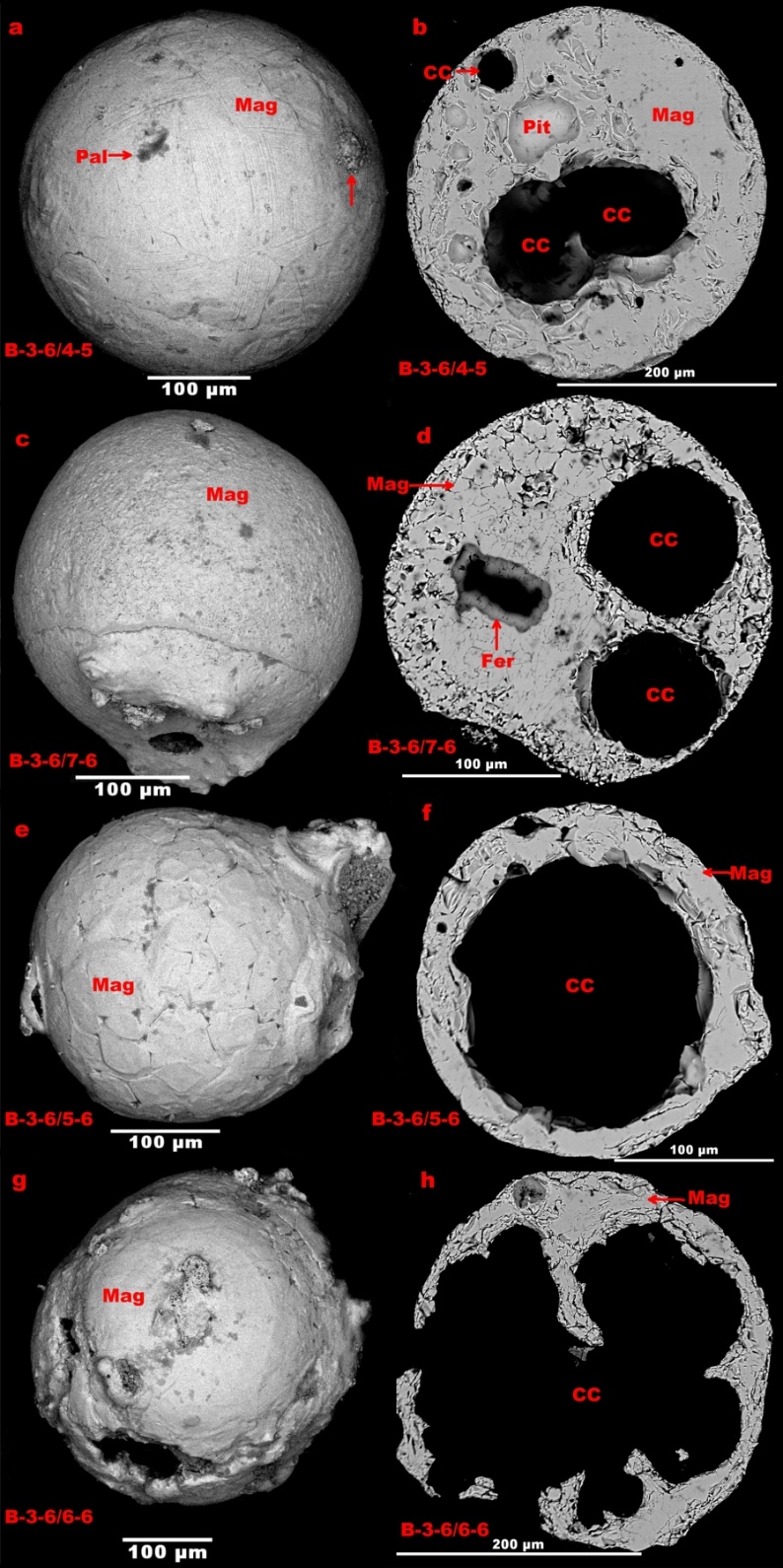


**Figure S2:** Magnetite spherules hosting cylindrical cavity. (a), (c), (e), and (g) showing the external surface of the spherules B-3-6/4-5, 7-6, 5-6, and 6-6, respectively. (b), (d), (f), and (h) showing the polished section of the spherule B-3-6/4-5, 7-6, 5-6, and 6-6, respectively. Here, Mag – Magnetite, Pal – Palagonite, CC – Cylindrical cavity.

**
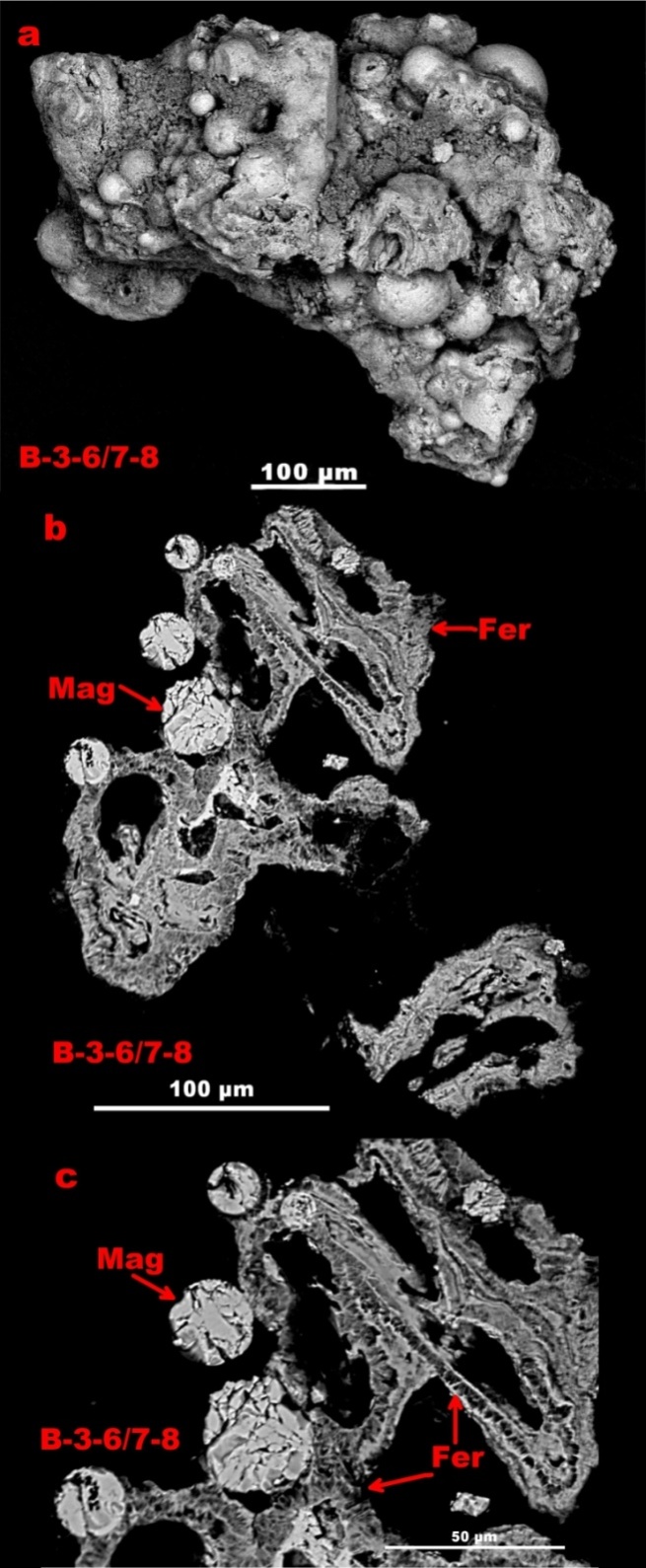
**

**Figure S3:** Multiple magnetite spherules fused by a Fe-oxide. (a) Several Fe-rich spherules embedded in the grain seem like blobs expelled outward, forming a spherical surface. The magnetite spherules have a smooth external surface. (b), (c) Shows the polished section of ‘a’, where the grain is mainly composed of a ferrihydrite matrix and magnetite spherules of different sizes. The magnetite spherules are composedof subhedral to anhedral magnetite minerals.

**
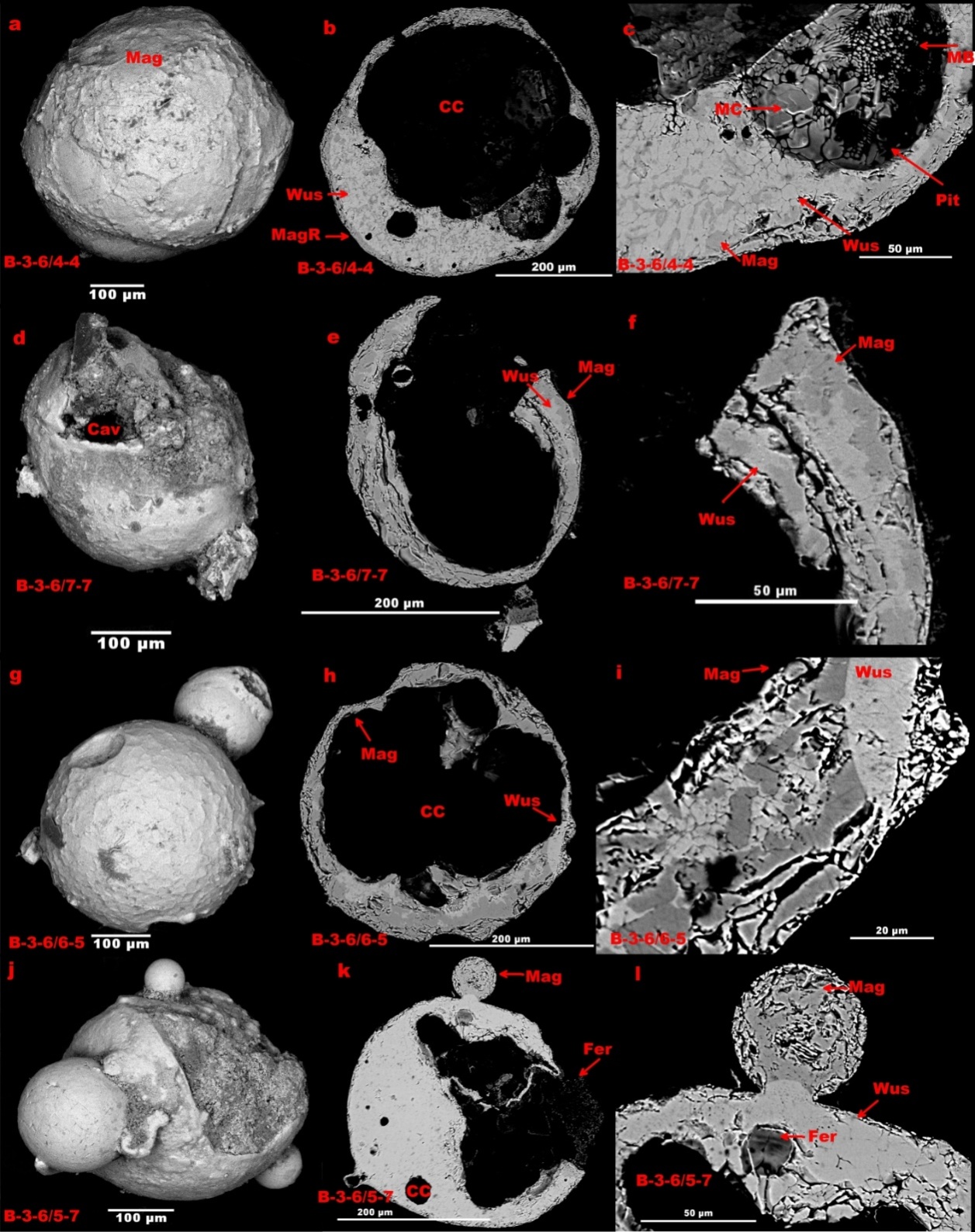
**

**Figure S4:** Wustite bearing spherules with the cylindrical cavity. (a), (d), (g), and (j) showing the external surface of the spherules B-3-6/4-4, 7-7, 6-5, and 5-7, respectively. (b), (c) showing the polished section of B-3-6/4-4. (e), (f) showing the polished section of B-3-6/7-7. (h), (i) showing the polished section of B-3-6/6-5. (k), (l) showing the polished section of B-3-6/5-7. Here, Mag – Magnetite, MagR – magnetite rim, Wus – wustite, CC – Cylindrical cavity, Cav – Cavity, Fer – Ferrihydrite, MC – Magnetite crystal, and MB – Magnetite bubble.

**
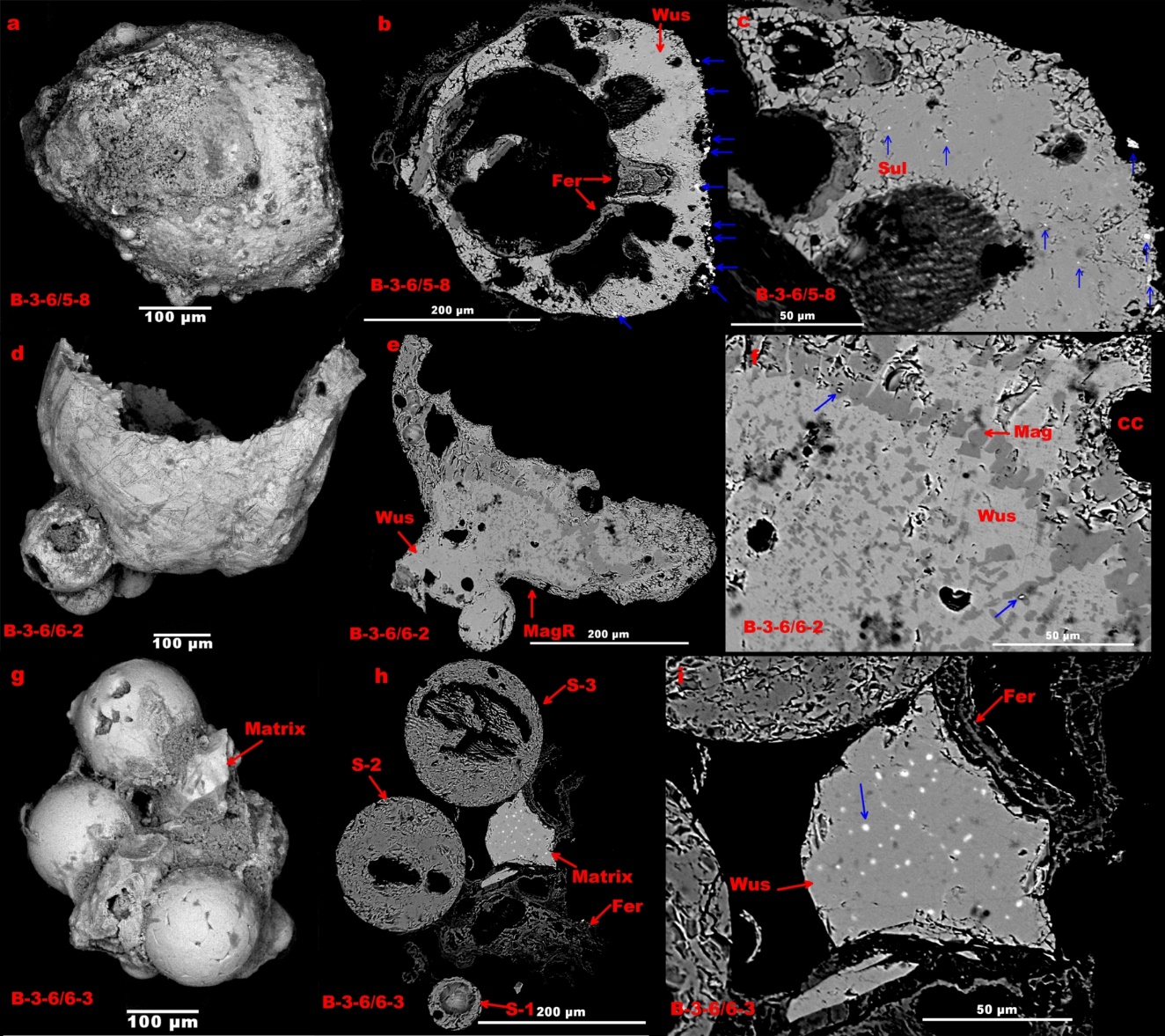
**

**Figure S5:** Spherules hosting sulfide nano-particles. (a), (d), and (g), showing the external surface of the spherules B-3-6/5-8, 6-2, and 6-3, respectively. (b), (c) showing the polished section of B-3-6/5-8. (e), (f) showing the polished section of B-3-6/6-2. (h), (i) showing the polished section of B-3-6/6-3. Here, Mag – Magnetite, MagR – Magnetite rim, Wus – wustite, CC – Cylindrical cavity, Fer – Ferrihydrite, and Sul – Sulfidenano-particle (shown by the blue arrow).

**
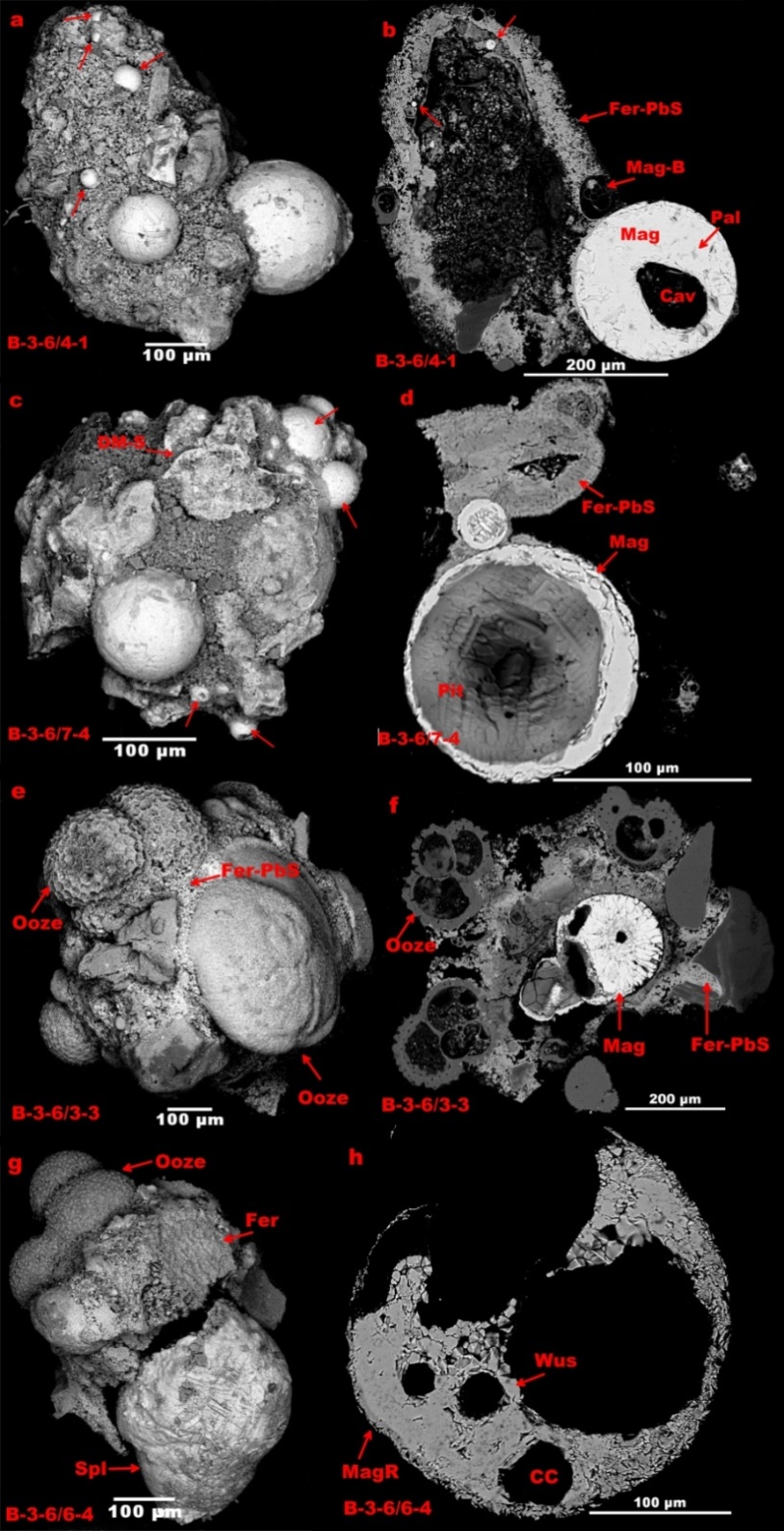
**

**Figure S6:** Spherules fused to sediments and oozes. (a), (c), (e), and (g) showing the external surface of the particles B-3-6/4-1, 7-4, 3-3, and 6-4, respectively. (b), (d), (f), and (h) showing the polished section of particles B-3-6/4-1, 7-4, 3-3, and 6-4, respectively. Here, smaller spherules are shown by the red arrow. Here, Mag – Magnetite, MagR – Magnetite rim, Mag-B – Magnetite bubble, Wus – wustite, CC – Cylindrical cavity, Cav – Cavity, spl – spherule, Fer – Ferrihydrite, DM-S – Dendritic matrix hosting sulfur, and Fer-PbS – Ferrihydrite hosting PbS (Galena).


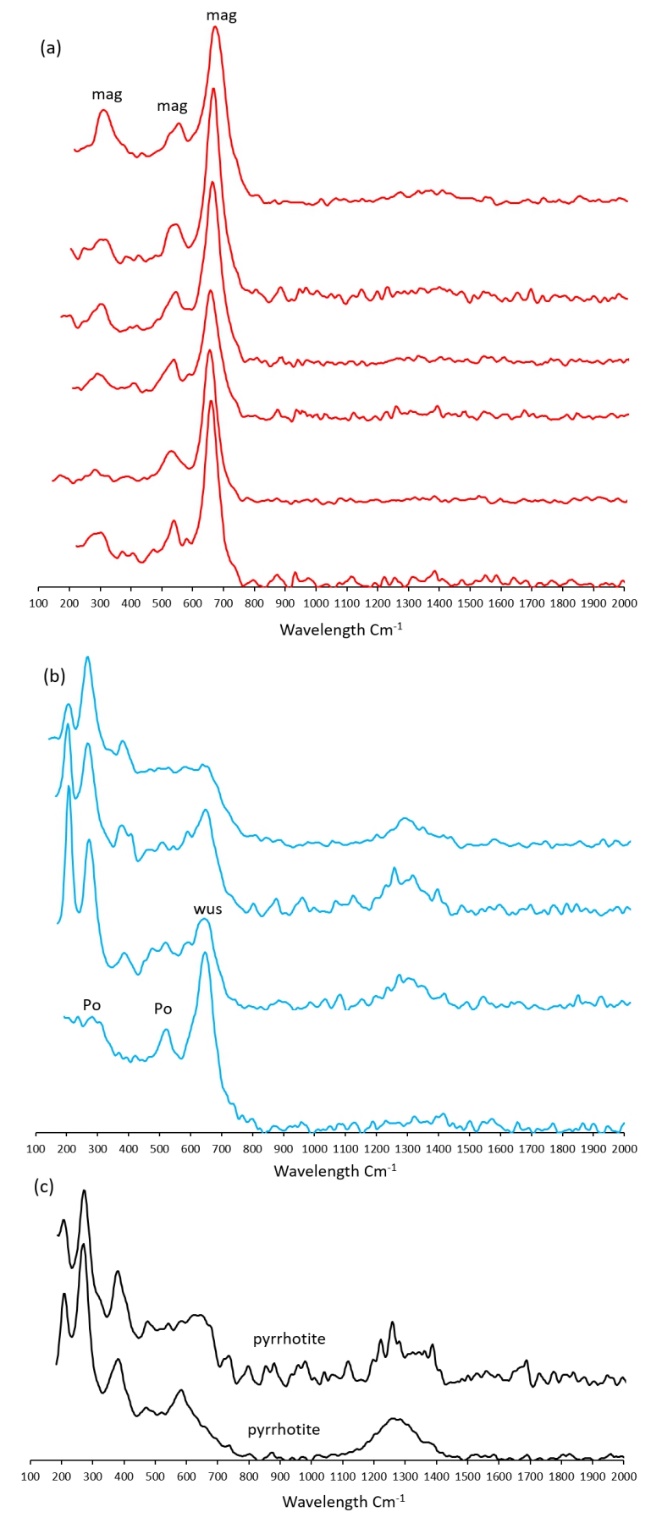


**Figure S7:** Raman Spectra collected on the polished surfaces of the spherules recovered from the CIR. (a) Magnetites(mag) produced similar spectra with absorption peaks near 660 Cm^-1^, 545 Cm^-1^, and 305 Cm^-1^. (b) Wustite (wus) produced similar spectra with absorption peaks near 650 Cm^-1^. (c) Pyrrhotite (Po) produced spectra with absorption peaks near 210 Cm^-1^, 275 Cm^-1^, 380 Cm^-1^, 525 Cm^-1^, 580 Cm^-1^, and 1280 Cm^-1^.

**Table S1**: Details of sample collection from CIR and SWIR, with dry sediment quantity processed and the number of spherules obtained from the sediment samples.

| . | Dredging started | | | Dredging stopped | | |  |  |  |  |
| --- | --- | --- | --- | --- | --- | --- | --- | --- | --- | --- |
| Sample no | Latitude South (DM) | Longitude East (DM) | Depth (m) | Latitude South (DM) | Longitude East (DM) | Depth (m) | Sediment | Initial Dry Weight (gm) | No. of spherules (>250 µ) | Spherule Density (No. of spherule/Kg) |
| B-3-6 | 24^o^49.16′ | 69^o^51.90′ | 3230 | 24^o^49.41′ | 69^o^51.28′ | 3000 | Fine grain sediment (Dark Green) | 84.7 | 19 | 224.32 |
| B-4-7 | 25^o^11.11′ | 70^o^00.10′ | 3181 | 25^o^10.65′ | 70^o^00.32′ | 3081 | Fine grain sediment (Brown) | 84.2 | 2 | 23.75 |
| DGR-B-04 | 25^o^12.05′ | 69^o^52.38′ | 3000 | 25^o^11.22′ | 69^o^51.85′ | 2400 | Muddy sediment | 43.7 | 2 | 45.77 |
| DGR-B-05 | 25^o^13.82′ | 69^o^49.93′ | 3000 | 25^o^15.08′ | 69^o^49.00′ | 2100 | Light coloured Sediment | 83.7 | 5 | 59.74 |

**Table S2:** Mineral chemistry results of the magnetite phase along with average values. Here, spl – spherule, mag – magnetite.

| Magnetite | Al | Si | P | Ca | Cr | S | Fe | Na | Mg | Co | Mn | Pb | O | Total |
| --- | --- | --- | --- | --- | --- | --- | --- | --- | --- | --- | --- | --- | --- | --- |
| 3-3 spl | 0.08 | 0.16 | - | - | - | - | 71.77 | - | - | 0.02 | 0.25 | - | 20.96 | 93.41 |
| 3-3 spl | 0.06 | - | - | - | - | - | 71.64 | - | - | - | 0.24 | - | 20.77 | 92.95 |
| 3-3 spl | 0.07 | - | - | - | - | - | 71.36 | - | - | - | 0.28 | - | 20.69 | 92.57 |
| 3-3 spl | 0.12 | 0.07 | - | - | - | - | 72.34 | - | - | - | 0.25 | - | 21 | 93.88 |
| 3-3 spl | 0.09 | 0.16 | - | - | - | - | 68.88 | - | - | - | 0.22 | - | 20.13 | 89.66 |
| 3-3 spl | 0.08 | 0.46 | - | - | - | - | 68.96 | - | - | 0.02 | 0.24 | - | 20.57 | 90.63 |
| Avg | 0.08 | 0.21 | - | - | - | - | 70.83 | - | - | 0.02 | 0.25 | - | 20.69 | 92.18 |
|  |  |  |  |  |  |  |  |  |  |  |  |  |  |  |
| 4-1 spl | 0.11 | 0.28 | 0.08 | - | - | - | 71.24 | - | - | 0.01 | 0.27 | - | 21.13 | 93.47 |
| 4-1 spl | 0.12 | - | - | - | - | - | 71.81 | - | - | - | 0.29 | - | 20.83 | 93.19 |
| 4-1 spl | 0.09 | - | - | - | - | - | 72.01 | - | - | - | 0.29 | - | 20.88 | 93.44 |
| 4-1 spl | 0.11 | 0.33 | - | - | - | - | 70.61 | - | - | 0.02 | 0.28 | - | 20.9 | 92.56 |
| 4-1 spl | 0.12 | 0.24 | - | - | - | - | 70.95 | - | - | - | 0.28 | - | 20.84 | 92.57 |
| 4-1 spl | 0.11 | 0.12 | - | - | - | - | 71.52 | - | - | - | 0.3 | - | 20.9 | 93.12 |
| 4-1 spl | 0.11 | 0.14 | - | - | - | - | 71.71 | - | - | 0.01 | 0.28 | - | 21 | 93.44 |
| Avg | 0.11 | 0.22 | 0.08 | - | - | - | 71.41 | - | - | 0.01 | 0.28 | - | 20.93 | 93.11 |
|  |  |  |  |  |  |  |  |  |  |  |  |  |  |  |
| 4-4 mag | 0.44 | 0.52 | - | - | - | 0.07 | 70.99 | - | - | - | 0.27 | - | 21.59 | 94.15 |
| 4-4 mag | 0.46 | 0.29 | - | - | - |  | 70.63 | - | - | 0.03 | 0.24 | - | 21.17 | 93 |
| 4-4 mag | 0.51 | 0.5 | - | 0.09 | - | 0.11 | 71.14 | - | - | - | 0.29 | - | 21.75 | 94.6 |
| 4-4 mag | 0.37 | 1.04 | - | 0.23 | - | 0.26 | 71.75 | - | - | - | 0.31 | - | 22.63 | 96.83 |
| 4-4 mag | 0.6 | 0.41 | - | - | - | 0.06 | 71.06 | - | - | - | 0.22 | - | 21.57 | 94.03 |
| Avg | 0.48 | 0.55 | - | 0.16 | - | 0.13 | 71.11 | - | - | 0.03 | 0.27 | - | 21.74 | 94.52 |
|  |  |  |  |  |  |  |  |  |  |  |  |  |  |  |
| 4-5 spl | - | - | - | - | - | - | 72 | - | - | 0.01 | 0.23 | - | 20.83 | 93.35 |
| 4-5 spl | - | 0.07 | - | - | - | - | 71.9 | - | - | 0.02 | 0.25 | - | 20.88 | 93.35 |
| 4-5 spl | - | 0.27 | 0.15 | - | - | - | 71.13 | - | - | 0.03 | 0.27 | - | 21.08 | 93.26 |
| 4-5 spl | 0.06 | 0.06 | - | - | - | - | 72.09 | - | - | 0.02 | 0.25 | - | 20.93 | 93.55 |
| 4-5 spl | - | 0.57 | - | - | - | - | 69.39 | 0.43 | - | - | 0.29 | - | 20.86 | 91.68 |
| 4-5 spl | 0.06 | - | - | - | 0.06 | - | 72.27 | - | - | 0.01 | 0.31 | - | 20.9 | 93.56 |
| 4-5 spl | - | - | - | - | - | - | 71.71 | - | - | - | 0.27 | - | 20.72 | 92.9 |
| 4-5 spl | - | 0.08 | - | - | - | - | 71.87 | - | - | - | 0.27 | - | 20.87 | 93.29 |
| 4-5 spl | - | 1.34 | - | - | - | - | 66.8 | 0.98 | - | 0.01 | 0.27 | - | 21.34 | 91.12 |
| 4-5 spl | - | 0.12 | - | - | - | - | 71.21 | - | - | - | 0.27 | - | 20.73 | 92.53 |
| Avg | 0.06 | 0.36 | 0.15 | - | - | - | 71.04 | 0.71 | - | 0.02 | 0.27 | - | 20.91 | 92.86 |
|  |  |  |  |  |  |  |  |  |  |  |  |  |  |  |
| 4-5 pit | - | 0.06 | - | - | - | - | 71.44 | - | - | 0.01 | 0.29 | - | 20.72 | 92.75 |
| 4-5 pit | - | 0.07 | - | - | - | - | 71.79 | - | - | - | 0.24 | - | 20.78 | 93.03 |
| 4-5 pit | - | 0.06 | - | - | - | - | 71.92 | - | - | - | 0.29 | - | 20.84 | 93.32 |
| 4-5 pit | 0.06 | 0.1 | - | - | - | - | 71.59 | - | - | - | 0.24 | - | 20.81 | 93.07 |
| Avg | 0.06 | 0.07 | - | - | - | - | 71.69 | - | - | 0.01 | 0.27 | - | 20.79 | 93.04 |
|  |  |  |  |  |  |  |  |  |  |  |  |  |  |  |
| 4-5 fade top | - | - | - | - | - | - | 71.11 | - | - | - | 0.23 | - | 20.56 | 92.09 |
| 4-5 fade top | - | - | - | - | - | - | 71.52 | - | - | - | 0.27 | - | 20.73 | 92.8 |
| Avg | - | - | - | - | - | - | 71.32 | - | - | - | 0.25 | - | 20.65 | 92.45 |
|  |  |  |  |  |  |  |  |  |  |  |  |  |  |  |
| 4-6 mag | - | 0.1 | - | - | - | - | 71.88 | - | - | 0.03 | 0.26 | - | 20.94 | 93.32 |
| 4-6 mag | - | - | - | - | - | - | 71.87 | - | - | - | 0.29 | - | 20.76 | 93.06 |
| 4-6 mag | - | - | - | - | - | - | 71.83 | - | - | 0.06 | 0.29 | - | 20.8 | 93.19 |
| 4-6 mag | - | 0.1 | - | - | - | - | 71.71 | - | - | - | 0.23 | - | 20.88 | 93.18 |
| 4-6 mag | - | - | - | - | - | - | 71.9 | - | - | - | 0.29 | - | 20.82 | 93.26 |
| 4-6 mag | - | - | - | - | - | - | 72.21 | - | - | - | 0.26 | - | 20.9 | 93.53 |
| Avg | - | 0.10 | - | - | - | - | 71.90 | - | - | 0.05 | 0.27 | - | 20.85 | 93.26 |
|  |  |  |  |  |  |  |  |  |  |  |  |  |  |  |
| 4-6 dark | 0.07 | 0.97 | - | - | - | - | 66.98 | 0.48 | - | 0.06 | 0.27 | - | 20.76 | 89.77 |
| 4-6 dark | - | 0.81 | 0.14 | - | - | - | 68.9 | - | - | 0.04 | 0.25 | - | 21.13 | 91.7 |
| 4-6 dark | 0.08 | 0.93 | - | - | - | - | 68.58 | 0.48 | - | 0.04 | 0.25 | - | 21.17 | 91.76 |
| 4-6 dark | - | 0.52 | - | - | - | - | 70.22 | - | - | - | 0.28 | - | 21.06 | 92.61 |
| 4-6 dark | 0.06 | 1.27 | 0.09 | - | - | - | 65.95 | 0.34 | - | - | 0.26 | - | 20.84 | 89.04 |
| Avg | 0.07 | 0.90 | 0.12 | - | - | - | 68.13 | 0.43 | - | 0.05 | 0.26 | - | 20.99 | 90.98 |
|  |  |  |  |  |  |  |  |  |  |  |  |  |  |  |
| 6-7 mag | - | 0.35 | 0.29 | - | - | - | 70.62 | - | - | 0.02 | 0.35 | - | 21.22 | 93.06 |
| 6-7 mag | - | - | - | - | - | - | 72.1 | - | - | 0.02 | 0.31 | - | 20.83 | 93.41 |
| 6-7 mag | - | 0.18 | 0.13 | - | - | - | 71.33 | - | - | - | 0.33 | - | 20.99 | 93.17 |
| 6-7 mag | - | 0.06 | - | - | - | - | 71.62 | - | - | - | 0.32 | - | 20.75 | 92.93 |
| 6-7 mag | - | 0.07 | - | - | - | - | 71.67 | - | - | - | 0.28 | - | 20.75 | 92.88 |
| 6-7 mag | - | 0.06 | - | - | - | - | 71.68 | - | - | - | 0.29 | - | 20.74 | 92.9 |
| Avg | - | 0.14 | 0.21 | - | - | - | 71.50 | - | - | 0.02 | 0.31 | - | 20.88 | 93.06 |
|  |  |  |  |  |  |  |  |  |  |  |  |  |  |  |
| 6-7 dark | - | 0.41 | 0.09 | - | - | - | 70.03 | - | - | - | 0.31 | - | 20.88 | 92.12 |
| 6-7 dark | - | 0.41 | 0.14 | 0.09 | - | - | 69.68 | - | 0.12 | 0.02 | 0.3 | - | 20.97 | 92.06 |
| 6-7 dark | - | 0.51 | - | - | - | - | 69.56 | 0.33 | - | 0.04 | 0.27 | - | 20.82 | 91.78 |
| Avg | - | 0.44 | 0.12 | 0.09 | - | - | 69.76 | 0.33 | 0.12 | 0.03 | 0.29 | - | 20.89 | 91.99 |
|  |  |  |  |  |  |  |  |  |  |  |  |  |  |  |
| 6-6 spl | 0.07 | 0.15 | - | - | - | - | 71.31 | - | - | - | 0.29 | - | 20.81 | 92.72 |
| 6-6 spl | 0.09 | 0.09 | - | - | - | - | 71.36 | - | - | 0.02 | 0.28 | - | 20.79 | 92.81 |
| 6-6 spl | 0.09 | 0.31 | - | - | - | - | 68.89 | - | 0.14 | 0.01 | 0.3 | - | 20.49 | 90.68 |
| 6-6 spl | 0.09 | 0.14 | - | - | - | - | 71.28 | - | - | - | 0.3 | - | 20.86 | 92.91 |
| 6-6 spl | 0.1 | 0.21 | 0.06 | - | - | - | 71.04 | - | - | 0.02 | 0.28 | - | 20.89 | 92.75 |
| Avg | 0.09 | 0.18 | 0.06 | - | - | - | 70.78 | - | 0.14 | 0.02 | 0.29 | - | 20.77 | 92.37 |
|  |  |  |  |  |  |  |  |  |  |  |  |  |  |  |
| 6-5 mag | 0.15 | - | - | - | - | - | 71.4 | - | - | 0.02 | 0.25 | - | 20.74 | 92.68 |
| 6-5 mag | 0.14 | - | 0.06 | - | - | - | 71.08 | - | - | - | 0.3 | - | 20.78 | 92.56 |
| 6-5 mag | 0.12 | - | - | - | - | - | 71.51 | - | - | 0.03 | 0.26 | - | 20.75 | 92.74 |
| 6-5 mag | 0.12 | 0.06 | - | - | - | - | 70.82 | - | - | 0.02 | 0.27 | - | 20.64 | 92.1 |
| 6-5 mag | 0.12 | - | - | - | - | - | 70.06 | - | - | - | 0.29 | - | 20.41 | 91.15 |
| Avg | 0.13 | 0.06 | 0.06 | - | - | - | 70.97 | - | - | 0.02 | 0.27 | - | 20.66 | 92.25 |
|  |  |  |  |  |  |  |  |  |  |  |  |  |  |  |
| 6-3 S1 pit | - | 0.15 | - | - | - | - | 71.09 | - | - | 0.02 | 0.33 | - | 20.71 | 92.5 |
|  |  |  |  |  |  |  |  |  |  |  |  |  |  |  |
| 6-3 S2 | - | - | 0.09 | - | - | - | 71.28 | - | - | 0.02 | 0.3 | - | 20.74 | 92.58 |
| 6-3 S2 | - | 0.13 | 0.08 | - | - | - | 71.45 | - | - | - | 0.31 | - | 20.88 | 92.97 |
| 6-3 S2 | - | - | - | - | - | - | 71.34 | - | - | - | 0.29 | - | 20.64 | 92.39 |
| 6-3 S2 | - | 0.08 | 0.1 | - | - | - | 70.98 | - | - | - | 0.26 | - | 20.7 | 92.32 |
| Avg | - | 0.11 | 0.09 | - | - | - | 71.26 | - | - | 0.02 | 0.29 | - | 20.74 | 92.57 |
|  |  |  |  |  |  |  |  |  |  |  |  |  |  |  |
| 6-3 S3 | - | 0.07 | - | - | - | - | 70.69 | - | - | 0.04 | - | - | 20.44 | 91.49 |
| 6-3 S3 | - | 0.08 | - | - | - | - | 70.83 | - | - | 0.03 | - | - | 20.47 | 91.59 |
| 6-3 S3 | - | 0.06 | - | - | - | - | 70.36 | - | - | - | - | - | 20.36 | 91.17 |
| 6-3 S3 | - | 0.07 | - | - | - | - | 70.01 | - | - | 0.01 | - | - | 20.25 | 90.64 |
| Avg | - | 0.07 | - | - | - | - | 70.47 | - | - | 0.03 | - | - | 20.38 | 91.22 |
|  |  |  |  |  |  |  |  |  |  |  |  |  |  |  |
| 6-2 mag | 0.06 | 0.53 | 0.17 | - | - | - | 70.77 | - | - | 0.01 | 0.32 | - | 21.42 | 93.69 |
| 6-2 mag | 0.1 | 0.07 | - | - | - | - | 71.51 | - | - | - | 0.24 | - | 20.74 | 92.71 |
| 6-2 mag | 0.06 | 0.08 | - | - | - | - | 72.9 | - | - | - | 0.31 | - | 21.18 | 94.62 |
| 6-2 mag | - | 0.11 | - | - | - | - | 72.15 | - | - | - | 0.24 | - | 20.98 | 93.66 |
| 6-2 mag | 0.07 | 0.08 | - | - | - | - | 72.37 | - | - | - | 0.25 | - | 21.02 | 93.9 |
| Avg | 0.07 | 0.17 | 0.17 | - | - | - | 71.94 | - | - | 0.01 | 0.27 | - | 21.07 | 93.72 |
|  |  |  |  |  |  |  |  |  |  |  |  |  |  |  |
| 7-4 spl | - | 0.1 | - | 0.13 | - | - | 70.73 | - | - | 0.03 | 0.2 | - | 20.53 | 91.85 |
|  |  |  |  |  |  |  |  |  |  |  |  |  |  |  |
| 7-5 mag | 0.12 | 0.15 | - | - | - | - | 71.24 | - | - | - | 0.26 | - | 20.83 | 92.69 |
| 7-5 mag | 0.09 | 0.29 | - | - | - | - | 71.2 | - | - | - | 0.28 | - | 20.99 | 93.06 |
| 7-5 mag | 0.09 | 0.98 | 0.11 | - | - | - | 69.75 | - | - | 0.04 | 0.29 | - | 21.48 | 92.92 |
| 7-5 mag | 0.11 | 0.22 | - | - | - | - | 70.79 | - | - | 0.01 | 0.26 | - | 20.78 | 92.33 |
| 7-5 mag | 0.1 | 0.3 | - | - | - | - | 71.1 | - | - | - | 0.28 | - | 20.97 | 92.85 |
| 7-5 mag | 0.11 | 0.31 | - | - | - | - | 70.32 | - | - | - | 0.27 | - | 20.8 | 92.04 |
| 7-5 mag | 0.12 | - | - | - | - | - | 71.26 | - | - | 0.01 | 0.25 | - | 20.7 | 92.56 |
| Avg | 0.11 | 0.38 | 0.11 | - | - | - | 70.81 | - | - | 0.02 | 0.27 | - | 20.94 | 92.64 |
|  |  |  |  |  |  |  |  |  |  |  |  |  |  |  |
| 7-5 dark | 0.1 | 1.23 | 0.19 | 0.19 | - | - | 68.76 | - | - | 0.02 | 0.28 | - | 21.66 | 92.55 |
| 7-5 dark | 0.09 | 0.52 | 0.08 | - | - | - | 70.51 | - | - | 0.07 | 0.28 | - | 21.12 | 92.77 |
| 7-5 dark | 0.12 | 0.38 | - | - | - | - | 70.77 | - | - | 0.02 | 0.25 | - | 21 | 92.76 |
| Avg | 0.10 | 0.71 | 0.14 | 0.19 | - | - | 70.01 | - | - | 0.04 | 0.27 | - | 21.26 | 92.69 |
|  |  |  |  |  |  |  |  |  |  |  |  |  |  |  |
| 7-6 mag | 0.11 | 0.15 | - | - | - | - | 71.09 | - | - | 0.01 | 0.28 | - | 20.84 | 92.77 |
| 7-6 mag | 0.09 | 0.47 | - | - | - | - | 68.63 | - | - | - | 0.28 | - | 20.47 | 90.14 |
| 7-6 mag | 0.09 | 0.66 | - | - | - | - | 70.25 | - | 0.06 | - | 0.26 | - | 21.12 | 92.64 |
| 7-6 mag | 0.09 | 0.61 | 0.1 | 0.17 | - | - | 69.79 | - | - | - | 0.29 | - | 21.1 | 92.27 |
| 7-6 mag | 0.09 | 0.42 | 0.08 | 0.14 | - | - | 70.42 | - | - | 0.03 | 0.26 | - | 21.04 | 92.71 |
| 7-6 mag | 0.09 | 0.66 | 0.09 | 0.14 | - | - | 68.05 | - | - | 0.01 | 0.27 | - | 20.73 | 90.51 |
| 7-6 mag | 0.1 | 0.32 | - | - | - | - | 70.62 | - | - | - | 0.28 | - | 20.92 | 92.56 |
| 7-6 mag | 0.1 | 0.47 | 0.08 | 0.12 | - | - | 70.64 | - | - | 0.01 | 0.27 | - | 21.18 | 93.14 |
| 7-6 mag | 0.1 | 0.56 | 0.1 | 0.14 | - | - | 70.4 | - | - | 0.02 | 0.28 | - | 21.24 | 93.01 |
| 7-6 mag | 0.1 | 0.67 | 0.1 | 0.15 | - | - | 68.16 | - | - | 0.05 | 0.24 | - | 20.77 | 90.54 |
| Avg | 0.10 | 0.50 | 0.09 | 0.14 | - | - | 69.81 | - | 0.06 | 0.02 | 0.27 | - | 20.94 | 92.03 |
|  |  |  |  |  |  |  |  |  |  |  |  |  |  |  |
| 7-6 zoned | 0.1 | 0.35 | 0.06 | - | - | - | 68.91 | - | - | 0.02 | 0.25 | - | 20.52 | 90.52 |
| 7-6 zoned | 0.08 | 0.39 | - | - | - | - | 67.7 | - | - | 0.03 | 0.31 | - | 20.19 | 89.09 |
| 7-6 zoned | 0.09 | 0.64 | 0.1 | 0.15 | - | - | 67.8 | - | - | - | 0.27 | - | 20.6 | 89.95 |
| Avg | 0.09 | 0.46 | 0.08 | 0.15 | - | - | 68.14 | - | - | 0.03 | 0.28 | - | 20.44 | 89.85 |
|  |  |  |  |  |  |  |  |  |  |  |  |  |  |  |
| 7-7 mag | 0.19 | 0.43 | 0.08 | - | - | - | 71.2 | - | - | - | 0.17 | - | 21.3 | 93.62 |
| 7-7 mag | 0.26 | 0.46 | 0.08 | - | - | - | 70.49 | - | - | 0.02 | 0.23 | - | 21.27 | 93.25 |
| 7-7 mag | 0.09 | 0.63 | 0.09 | - | - | - | 71.28 | - | - | 0.01 | 0.28 | 0.49 | 21.59 | 94.77 |
| 7-7 mag | 0.21 | 0.49 | 0.07 | - | - | - | 71.66 | - | - | 0.03 | 0.25 | - | 21.56 | 94.65 |
| Avg | 0.19 | 0.50 | 0.08 | - | - | - | 71.16 | - | - | 0.02 | 0.23 | 0.49 | 21.43 | 94.07 |
|  |  |  |  |  |  |  |  |  |  |  |  |  |  |  |
| 7-8 spl | - | 0.21 | - | - | - | - | 69.49 | - | - | - | 0.18 | - | 20.31 | 90.32 |
| 7-8 spl | - | 0.09 | - | - | - | - | 71.65 | - | - | 0.04 | 0.25 | - | 20.8 | 93.01 |
| 7-8 spl | - | 0.42 | - | - | - | - | 69.72 | - | - | 0.01 | 0.17 | - | 20.65 | 91.24 |
| Avg | - | 0.24 | - | - | - | - | 70.29 | - | - | 0.03 | 0.20 | - | 20.59 | 91.52 |
|  |  |  |  |  |  |  |  |  |  |  |  |  |  |  |
| 5-6 spl | - |  | - | - | - | - | 72.15 | - | - | - | 0.27 | - | 20.84 | 93.41 |
| 5-6 spl | - | 0.06 | - | - | - | - | 72.42 | - | - | - | 0.31 | - | 21 | 93.95 |
| 5-6 spl | - | 0.11 | - | - | - | - | 69.41 | - | - | - | 0.23 | - | 20.16 | 90.04 |
| 5-6 spl | 0.39 |  | - | - | - | - | 71.49 | - | - | 0.01 | 0.3 | - | 21.06 | 93.43 |
| 5-6 spl | - |  | - | - | - | - | 72.21 | - | - | 0.03 | 0.3 | - | 20.92 | 93.6 |
| 5-6 spl | - |  | - | - | - | - | 70.56 | - | - | - | 0.28 | - | 20.4 | 91.38 |
| 5-6 spl | 0.62 | 0.07 | - | - | - | - | 69.78 | - | - | - | 0.29 | - | 20.79 | 91.73 |
| Avg | 0.51 | 0.08 | - | - | - | - | 71.15 | - | - | 0.02 | 0.28 | - | 20.74 | 92.51 |
|  |  |  |  |  |  |  |  |  |  |  |  |  |  |  |
| 5-7 mag | - | 0.13 | - | - | - | - | 70.97 | - | - | 0.05 | 0.28 | - | 20.7 | 92.35 |
| 5-7 mag | - | 0.17 | - | - | - | - | 70.27 | - | - | 0.02 | 0.27 | - | 20.57 | 91.59 |
| 5-7 mag | - | 0.19 | - | - | - | - | 70.31 | - | - | 0.02 | 0.24 | - | 20.56 | 91.5 |
| 5-7 mag | - | 0.25 | - | - | - | - | 69.88 | - | - | 0.04 | 0.26 | - | 20.58 | 91.46 |
| Avg | - | 0.19 | - | - | - | - | 70.36 | - | - | 0.03 | 0.26 | - | 20.60 | 91.73 |

**Table S3:** The mineral chemistry results of the wustite phase along with average values. Here, wus – wustite.

| Wustite | Al | Si | P | Ca | S | Fe | Mg | Co | Mn | O | Total |
| --- | --- | --- | --- | --- | --- | --- | --- | --- | --- | --- | --- |
| 4-4 wus | 0.35 | 0.4 | - | - | 0.07 | 72.98 | - | 0.01 | 0.28 | 21.99 | 96.47 |
| 4-4 wus | 0.31 | 0.44 | - | - | 0.15 | 73.25 | - | 0 | 0.32 | 22.09 | 96.76 |
| 4-4 wus | 0.28 | 0.12 | - | - | 0.03 | 74.92 | - | 0.01 | 0.31 | 22.03 | 97.88 |
| 4-4 wus | 0.28 | 0.13 | - | - | - | 74.99 | - | 0.02 | 0.3 | 22.08 | 97.96 |
| 4-4 wus | 0.17 | 0.9 | - | 0.17 | 0.19 | 73.28 | - | 0.01 | 0.35 | 22.66 | 98 |
| Avg | 0.28 | 0.40 | - | 0.17 | 0.11 | 73.88 | - | 0.01 | 0.31 | 22.17 | 97.41 |
|  |  |  |  |  |  |  |  |  |  |  |  |
| 4-6 wus | - | - | - | - | - | 74.77 | - | - | 0.31 | 21.64 | 96.96 |
| 4-6 wus | - | 0.08 | - | - | - | 74.83 | - | - | 0.27 | 21.72 | 97.08 |
| 4-6 wus | - | 0.18 | 0.09 | - | - | 74.36 | - | - | 0.28 | 21.79 | 96.86 |
| 4-6 wus | - | 0.08 | - | - | - | 74.41 | - | 0.07 | 0.29 | 21.65 | 96.81 |
| Avg | - | 0.11 | 0.09 | - | - | 74.59 | - | 0.07 | 0.29 | 21.70 | 96.93 |
|  |  |  |  |  |  |  |  |  |  |  |  |
| 6-5 wus | - | 0.11 | 0.22 | - | - | 73.63 | - | 0.01 | 0.36 | 21.78 | 96.52 |
| 6-5 wus | 0.12 | 0.11 | - | - | 0.51 | 73.37 | - | - | 0.27 | 21.88 | 96.34 |
| 6-5 wus | 0.12 | - | - | - | - | 73.94 | - | - | 0.3 | 21.48 | 95.94 |
| Avg | 0.12 | 0.11 | 0.22 | - | 0.51 | 73.65 | - | 0.01 | 0.31 | 21.71 | 96.27 |
|  |  |  |  |  |  |  |  |  |  |  |  |
| 6-4 wus | - | 0.06 | - | - | - | 74.03 | - | - | 0.39 | 21.51 | 96.22 |
| 6-4 wus | - | - | - | - | - | 73.97 | - | - | 0.33 | 21.44 | 95.96 |
| 6-4 wus | - | 0.13 | - | - | - | 73.29 | - | 0.04 | 0.34 | 21.4 | 95.42 |
| 6-4 wus | - | 0.06 | - | - | - | 74.41 | - | 0.01 | 0.36 | 21.6 | 96.61 |
| 6-4 wus | - | - | - | - | - | 73.98 | - | - | 0.39 | 21.45 | 96.09 |
| Avg | - | 0.08 | - | - | - | 73.94 | - | 0.03 | 0.36 | 21.48 | 96.06 |
|  |  |  |  |  |  |  |  |  |  |  |  |
| 6-3 wus | - | 0.36 | 0.28 | - | 0.75 | 73.27 | - | 0.03 | 0.55 | 22.8 | 98.36 |
| 6-3 wus | - | 0.13 | 0.06 | - | 0.52 | 75.01 | - | 0.04 | 0.56 | 22.44 | 98.74 |
| 6-3 wus | - | 0.12 | - | - | 0.3 | 75.21 | - | 0.01 | 0.59 | 22.24 | 98.61 |
| 6-3 wus | - | - | - | - | - | 75.71 | - | - | 0.58 | 21.93 | 98.33 |
| Avg | - | 0.20 | 0.17 | - | 0.52 | 74.80 | - | 0.03 | 0.57 | 22.35 | 98.51 |
|  |  |  |  |  |  |  |  |  |  |  |  |
| 6-3 bright spot | - | 0.2 | 0.16 | - | 0.28 | 75.86 | - | 0.01 | 0.58 | 22.65 | 99.81 |
| 6-3 bright spot | - | 0.14 | - | - | 0.08 | 75.4 | - | - | 0.6 | 22.11 | 98.46 |
| 6-3 bright spot | - | 0.19 | 0.11 | - | 0.77 | 74.58 | - | - | 0.56 | 22.7 | 99.06 |
| Avg | - | 0.18 | 0.14 | - | 0.38 | 75.28 | - | 0.01 | 0.58 | 22.49 | 99.11 |
|  |  |  |  |  |  |  |  |  |  |  |  |
| 6-2 wus | - | 0.1 | - | - | 0.15 | 75.35 | - | 0.01 | 0.4 | 22.08 | 98.24 |
| 6-2 wus | - | 0.1 | 0.09 | - | 0.23 | 74.82 | - | 0.01 | 0.33 | 22.03 | 97.74 |
| 6-2 wus | 0.12 | 0.17 | 0.08 | - | 0.13 | 74.68 | - | 0.01 | 0.36 | 22.05 | 97.7 |
| 6-2 wus | 0.07 | 0.2 | 0.06 | - | 0.06 | 74.33 | - | - | 0.25 | 21.84 | 96.93 |
| Avg | 0.10 | 0.14 | 0.08 | - | 0.14 | 74.80 | - | 0.01 | 0.34 | 22.00 | 97.65 |
|  |  |  |  |  |  |  |  |  |  |  |  |
| 7-7 wus | 0.12 | 0.46 | 0.09 | - | - | 73.34 | - | 0.01 | 0.28 | 22 | 96.78 |
| 7-7 wus | 0.08 | 0.3 | 0.07 | - | - | 73.46 | 0.1 | - | 0.27 | 21.75 | 96.28 |
| 7-7 wus | - | 0.07 | - | - | - | 72.97 | - | 0.03 | 0.59 | 21.27 | 95.15 |
| Avg | 0.10 | 0.28 | 0.08 | - | - | 73.26 | 0.10 | 0.02 | 0.38 | 21.67 | 96.07 |
|  |  |  |  |  |  |  |  |  |  |  |  |
| 5-7 wus | - | 0.08 |  | - | 0.15 | 75.18 | - | 0.01 | 0.3 | 22.03 | 98.05 |
| 5-7 wus | - |  |  | - |  | 75.52 | - | 0.05 | 0.39 | 21.88 | 98.03 |
| 5-7 wus | - | 0.08 | - | - | - | 75.27 | - | 0.01 | 0.33 | 21.93 | 97.86 |
| 5-7 wus | - | 0.15 | - | - | 0.11 | 74.53 | - | - | 0.29 | 21.83 | 97.1 |
| 5-7 wus | - | 0.16 | 0.17 | - | - | 74.7 | - | 0.04 | 0.32 | 22.15 | 97.88 |
| 5-7 wus | - | - | - | - | - | 75.33 | - | - | 0.36 | 21.86 | 97.79 |
| Avg | - | 0.12 | 0.17 | - | 0.13 | 75.09 | - | 0.03 | 0.33 | 21.95 | 97.79 |
|  |  |  |  |  |  |  |  |  |  |  |  |
| 5-8 wus | 0.07 | 0.26 | - | - | 0.19 | 74.34 | - | - | 0.48 | 22.08 | 97.55 |
| 5-8 wus | 0.09 | 0.32 | - | - | 0.47 | 74.21 | - | - | 0.47 | 22.4 | 98.09 |
| 5-8 wus | 0.11 | 0.51 | - | - | 0.36 | 73.87 | - | 0.01 | 0.47 | 22.45 | 97.97 |
| 5-8 wus | 0.1 | 0.22 | - | - | 0.13 | 74.4 | - | - | 0.48 | 22.02 | 97.53 |
| 5-8 wus | 0.13 | 0.45 | - | - | 0.48 | 72.88 | - | - | 0.44 | 22.22 | 96.73 |
| Avg | 0.10 | 0.35 | - | - | 0.33 | 73.94 | - | 0.01 | 0.47 | 22.23 | 97.57 |

**Table S4:** Mineral chemistry results of ferrihydrite, carbonate, and other phases along with average values. Here, spl – spherule, fer - ferrihydrite.

| Element | Al | Si | P | K | Ca | Ti | Cr | S | Fe | V | Na | Mg | Co | Mn | Pb | O | Total |
| --- | --- | --- | --- | --- | --- | --- | --- | --- | --- | --- | --- | --- | --- | --- | --- | --- | --- |
| 3-3 carbonate | 0.03 | 0.72 | 0.12 | - | 35.04 | - | - | 0.06 | 0.85 | - | 0.28 | 0.1 | - | - | - | 15.45 | 52.71 |
| 3-3 carbonate | - | 0.13 | 0.11 | - | 35.42 | - | - | 0.11 | 0.53 | - | 0.33 | 0.09 | - | - | - | 14.86 | 51.6 |
| 3-3 carbonate | - | 0.11 | 0.1 | - | 32.48 | - | - | 0.06 | 0.42 | - | - | 0.08 | - | - | - | 13.54 | 47.07 |
| 3-3 carbonate | - | 0.47 | 0.13 | - | 36.29 | - | - | 0.05 | 2.78 | - | - | 0.14 | - | 0.02 | - | 16.22 | 56.4 |
| Avg | 0.03 | 0.36 | 0.12 | - | 34.81 | - | - | 0.07 | 1.15 | - | 0.31 | 0.10 | - | 0.02 | - | 15.02 | 51.95 |
|  |  |  |  |  |  |  |  |  |  |  |  |  |  |  |  |  |  |
| 3-3 fer | 0.16 | 5.34 | 0.12 | - | 0.64 | - | - | - | 46.86 | - | - | 0.77 | - | - | 0.76 | 20.83 | 76.02 |
| 3-3 fer | 0.33 | 8.28 | 0.29 | - | 1.2 | 0.27 | - | 0.07 | 44.22 | - | 0.61 | 1.13 | 0.02 | - | 1.12 | 24.66 | 82.62 |
| 3-3 fer | 0.4 | 12.57 | 0.5 | 0.24 | 1.1 | 0.26 | - | 0.06 | 30.17 | - | 1.25 | 1.54 | 0.03 | - | - | 26.24 | 74.73 |
| 3-3 fer | 0.07 | 6 | 0.37 | - | 2.7 | - | - | 0.07 | 45.5 | - | 0.28 | 0.69 | - | - | 1.71 | 22.33 | 80.01 |
| Avg | 0.24 | 8.05 | 0.32 | 0.24 | 1.41 | 0.27 | - | 0.07 | 41.69 | - | 0.71 | 1.03 | 0.03 | - | 1.20 | 23.52 | 78.35 |
|  |  |  |  |  |  |  |  |  |  |  |  |  |  |  |  |  |  |
| 4-1 fer | 0.19 | 7.16 | 0.19 | - | 0.91 | - | - | - | 43.18 | - | - | 2.94 | 0.02 | - | 0.86 | 23.45 | 79.3 |
| 4-1 fer | 0.1 | 5.33 | 0.14 | - | 0.67 | - | - | - | 48.24 | - | - | 1.98 | - | - | 0.83 | 21.98 | 79.77 |
| 4-1 fer | 0.08 | 4.18 | 0.15 | - | 0.87 | - | - | - | 46.24 | - | - | 0.42 | - | - | 0.31 | 19.1 | 71.79 |
| 4-1 fer | - | 4.26 | 0.13 | - | 0.73 | - | - | - | 51.79 | - | - | 0.71 | 0.01 | - | 0.32 | 20.84 | 79.27 |
| 4-1 fer | 0.27 | 8.15 | 0.31 | - | 1.33 | - | - | 0.06 | 43.78 | - | 0.38 | 0.64 | 0.02 | - | 0.69 | 23.74 | 79.63 |
| Avg | 0.16 | 5.82 | 0.18 | - | 0.90 | - | - | 0.06 | 46.65 | - | 0.38 | 1.34 | 0.02 | - | 0.60 | 21.82 | 77.95 |
|  |  |  |  |  |  |  |  |  |  |  |  |  |  |  |  |  |  |
| 4-4 bubble | 0.13 | 0.37 | - | - | - | - | - | - | 63.05 | - | - | - | 0.02 | 0.27 | - | 18.89 | 83.19 |
| 4-4 bubble | 0.12 | 0.37 | - | - | - | - | - | 0.06 | 60.26 | - | - | - | - | 0.26 | - | 17.99 | 79.22 |
| 4-4 bubble | 0.2 | 0.55 | - | - | - | - | - | 0.06 | 62.46 | - | - | - | 0.03 | 0.28 | - | 19.02 | 83.07 |
| Avg | 0.15 | 0.43 | - | - | - | - | - | 0.06 | 61.92 | - | - | - | 0.03 | 0.27 | - | 18.63 | 81.83 |
|  |  |  |  |  |  |  |  |  |  |  |  |  |  |  |  |  |  |
| 6-3 fer | 0.08 | 0.93 | 0.08 | - | 0.29 | - | - | - | 55.22 | - | - | 0.23 | - | 0.24 | 1.00 | 17.66 | 76.21 |
| 6-3 fer | - | 0.06 | - | - | - | - | - | - | 61.44 | - | - | - | - | 0.29 | - | 17.82 | 79.74 |
| Avg | 0.08 | 0.50 | 0.08 | - | 0.29 | - | - | - | 58.33 | - | - | 0.23 | - | 0.27 | 1.00 | 17.74 | 77.98 |
|  |  |  |  |  |  |  |  |  |  |  |  |  |  |  |  |  |  |
| 7-4 Small spl | - | 0.15 | - | - | - | - | - | - | 67.25 | - | - | - | 0.02 | 0.18 | - | 19.55 | 87.21 |
|  |  |  |  |  |  |  |  |  |  |  |  |  |  |  |  |  |  |
| 7-4 fer | - | 0.89 | 0.19 | - | 0.35 | - | - | 0.06 | 61.65 | - | - | 0.24 | 0.05 | 0.16 | - | 19.44 | 83.39 |
| 7-4 fer | - | 1.42 | 0.32 | - | 0.5 | - | - | 0.07 | 57.88 | - | - | 0.3 | 0.02 | 0.12 | - | 19.23 | 80.28 |
| 7-4 fer | 0.06 | 1.49 | 0.28 | - | 0.56 | - | 0.03 | 0.1 | 55.71 | 0.06 | - | 0.41 | - | 0.22 | 0.37 | 18.87 | 78.39 |
| Avg | 0.06 | 1.27 | 0.26 | - | 0.47 | - | 0.03 | 0.08 | 58.41 | 0.06 | - | 0.32 | 0.04 | 0.17 | 0.37 | 19.18 | 80.69 |
|  |  |  |  |  |  |  |  |  |  |  |  |  |  |  |  |  |  |
| 7-6 zoned | 0.08 | 0.46 | 0.06 | - | 0.15 | - | - | - | 64.68 | - | - | - | 0.02 | 0.26 | - | 19.43 | 85.36 |
| 7-6 zoned | 0.09 | 0.39 | 0.06 | - | 0.13 | - | - | - | 66.84 | - | - | - | 0.03 | 0.26 | - | 19.98 | 88.07 |
| 7-6 zoned | 0.09 | 0.64 | 0.11 | - | 0.19 | - | - | - | 66.67 | - | - | - | - | 0.28 | - | 20.32 | 88.61 |
| Avg | 0.09 | 0.50 | 0.08 | - | 0.16 | - | - | - | 66.06 | - | - | - | 0.03 | 0.27 | - | 19.91 | 87.35 |
|  |  |  |  |  |  |  |  |  |  |  |  |  |  |  |  |  |  |
| 7-6 zoned middle | 0.08 | 0.36 | - | - | 0.1 | - | - | 0.03 | 57.72 | - | - | - | 0.04 | 0.24 | - | 17.32 | 76.17 |
| 7-6 zoned middle | 0.08 | 0.26 | - | - | - | - | - | 0.03 | 55.35 | - | - | - | 0.01 | 0.23 | - | 16.5 | 72.79 |
| 7-6 zoned middle | 0.09 | 0.25 | - | - | - | - | - | - | 51.87 | - | - | 0.06 | - | 0.22 | - | 15.48 | 68.31 |
| 7-6 zoned middle | 0.09 | 0.25 | - | - | - | - | - | - | 50.94 | - | - | - | - | 0.17 | - | 15.2 | 66.99 |
| 7-6 zoned middle | 0.09 | 0.26 | - | - | 0.09 | - | - | - | 58.87 | - | - | - | - | 0.24 | - | 17.51 | 77.3 |
| 7-6 zoned middle | 0.09 | 0.42 | 0.08 | - | 0.12 | - | - | - | 57.71 | - | - | - | 0.02 | 0.21 | - | 17.4 | 76.29 |
| 7-6 zoned middle | 0.1 | 0.38 | - | - | 0.09 | - | - | 0.03 | 56.63 | - | - | - | - | 0.22 | - | 16.97 | 74.55 |
| 7-6 zoned middle | 0.08 | 0.3 | 0.07 | - | 0.1 | - | - | - | 55.71 | - | - | - | 0.01 | 0.23 | - | 16.65 | 73.32 |
| Avg | 0.09 | 0.31 | 0.08 | - | 0.10 | - | - | 0.03 | 55.60 | - | - | 0.06 | 0.02 | 0.22 | - | 16.63 | 73.22 |
|  |  |  |  |  |  |  |  |  |  |  |  |  |  |  |  |  |  |
| 7-8 fer | 0.09 | 1.86 | 0.16 | - | 0.5 | - | - | 0.14 | 49.7 | - | - | 0.29 | 0.03 | - | - | 17.36 | 70.71 |
| 7-8 fer | - | 1.56 | 0.15 | - | 0.42 | - | - | 0.06 | 60.32 | - | - | 0.23 | - | 0.19 | - | 19.81 | 83.12 |
| Avg | 0.09 | 1.71 | 0.16 | - | 0.46 | - | - | 0.10 | 55.01 | - | - | 0.26 | 0.03 | 0.19 | - | 18.59 | 76.92 |
